# A Computational Study of Photoinduced Borylation for Selected Boron Sources

**DOI:** 10.1002/open.202300285

**Published:** 2024-03-08

**Authors:** Ka Wa Fan, Hoi Ling Luk, David Lee Phillips

**Affiliations:** ^1^ Department of Chemistry The University of Hong Kong Hong Kong P. R. China

**Keywords:** EDA complex, Borylation, B_2_cat_2_

## Abstract

This research article uses density functional theory (DFT) to study photoinduced borylation. This work examined the electron donor‐acceptor complex (EDA) of bis(catecholato)diboron with different redox‐active leaving groups and bis(pinacol)diboron with aryl N‐hydroxyphthalimide. The results of these DFT studies show the complex ratio of B_2_cat_2_ and N, N‐dimethylacetamide (DMA) should be 1 : 2 which is consistent with the experimental results in the literature. We further proposed a reaction mechanism and calculated the energies associated with each step.

## Graphical abstract

The bis(catecholato)diboron(B_2_cat_2_) in dimethylacetamide (DMA) is popular boron source in photoinduced borylation. This article has studied the complex by DFT. It first compares the stoichiometry between the B_2_cat_2_ with 1 DMA and B_2_cat_2_ with 2 DMA. Based on the coordination of boron compound, two type of photoinduced borylation reaction mechanism were discussed and compared.

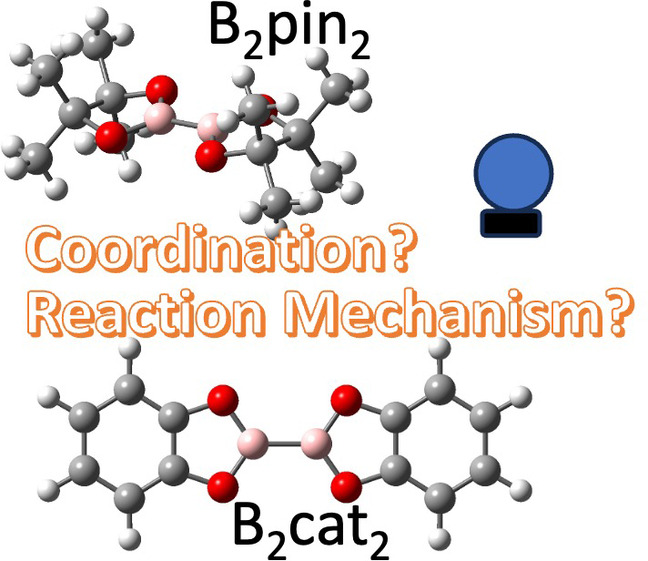

## Introduction

Borylation is an important reaction in organic synthesis, but it typically uses expensive materials such as palladium in the Miyaura borylation^[1]^ or iridium in photoredox catalyst.[^2^, ^3^, ^4^] Hence, a lot of research groups have tried to look for alternative ways to obtain borylation products.^[5]^ The light‐induced reaction would be one of the more desirable methods for borylation.^[6]^ This kind of reaction can be started with an aryl chloride,^[7]^ a photocatalyst[^8^, ^9^, ^10^, ^11^] and also an electron donor acceptor complex (EDA) approach.[^12^, ^13^, ^14^, ^15^, ^16^]

The EDA complex approach is not only limited to borylation, but also can be applied to a lot of different types of reactions.[^17^, ^18^, ^19^] EDA complex is a type of reaction where typically an additional absorption band will appear in the UV‐Visible spectrum. When this new absorption band is excited with an appropriate wavelength of light, the charge transfer from the donor to acceptor will take place. This will start the conversion process for some of the common and cheap functional groups to the redox active functional group of the reaction system. Some examples include the carboxylic acid conversion to the N‐hydroxyphthalimide ester (NHPI ester).^[20]^ the aromatic amine group conversion to an aryl diazonium tetrafluoroborate salt^[21]^ and the aliphatic amine conversion to Katritzky salts.^[22]^ Similar activation reactions can also occur for some EDA reaction systems.

**Scheme 1:**
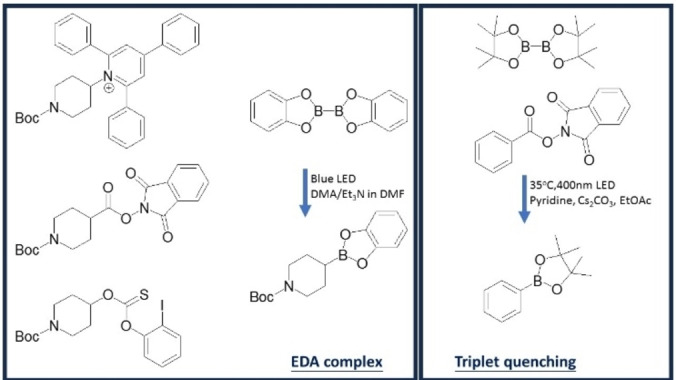
Four reactions studied in the paper.

In this paper, we mainly focused on four reactions for borylation. Glorius and co‐workers reported on an aryl N‐hydroxyphthalimide ester,^[23]^ Aggarwal and co‐workers reported on an aliphatic N‐hydroxyphthalimide ester,^[15]^ on a Katritzky salt[^12^, ^14^] and on 2‐iodophenyl‐thionocarbonates.^[13]^ They use bis(catecholato)diboron (B_2_cat_2_) to act as a donor in these literature reports. These approaches can also be applied to different systems.[^24^, ^25^, ^26^, ^27^, ^28^] These studies discussed the interactions between B_2_cat_2_ with DMA and had some discussion of the reaction mechanisms. The 4 reactions can be found in Scheme 1.

## Computational details

All the calculations here were performed in the Gaussian 16 program.^[29]^ The B3LYP‐D3BJ^[30]^ and smd solvation model^[31]^ were applied to all geometry optimizations. These computations used 6‐31 g(d) and 6‐31+g(d) for the anion to calculate the reaction between bis(pinacolato)diboron and aryl N‐hydroxyphthalimide ester. These calculations used 6‐31+g(d) for the reaction of bis(catecholato)diboron in dimethylacetamide. The reaction of bis(catecholato)diboron in dimethylformamide employed mixed basis sets. The mixed basis sets were SDD for iodine and 6‐311+g(d) for remaining elements. The transition state search utilized the Berny algorithm. It was confirmed that there is no imaginary frequency for the structures in the local minimums and only one imaginary frequency for the transition states.

For the NMR calculations, the GIAO method was used to calculate the isotropic value. The basis sets were pcSseg‐2^[32]^ and the source comes from the Basis Set Exchange.^[33]^ The Fukui function of the radical attack for some selected compounds are also presented.^[34]^ The NBO spin density was calculated by NBO 3.0 version.

The single energy point calculations were further calculated with DSDPBEP86 with 6‐31+g(d) (SDD pseudo potential for iodine) and compared with the differences between the different reactions examined here.

## Results and Discussion

To look at the interactions between the B_2_cat_2_ and ligands, the binding electronic and free energy of the different complexes were calculated and shown in Table 1. Most of the complex formations were determined to be exothermic in electronic energies. However, the boron complex with the 4‐picoline was an exception and other boron complexes were determined to be endergonic in the binding free energy. This indicates the binding process pays a high entropic cost.

**Table 1:** The binding energies of bis(catecholato)diboron with different ligands.

solvent	ligand	ratio	ΔE(kcal/mol)	ΔG(kcal/mol)
DMA	DMA	1 : 1	−7.8	5.6
		1 : 2	−14.2	14.9
CHCl_3_	4‐picoline	1 : 2	−30.1	−2.3
DMF	Et_3_N	1 : 2	−18.8	17.2

Another way to look at the interactions of B_2_cat_2_ and DMA can be found from the ^11^B NMR calculations. Aggarwal and coworkers had reported the ^11^B spectrum of the B_2_cat_2_ in CH_2_Cl_2_ and DMA solvents.^[15]^ They realized an additional peak at 13.8 ppm recorded in the DMA solvent. The chemical shift at 29.8 ppm in CHCl_3_ is also upfield to 25.4 ppm in the DMA solvent. They further compared the literature^[35]^ and deduced the B_2_cat_2_ with one DMA complex would be formed under these conditions.

**Figure 1:**
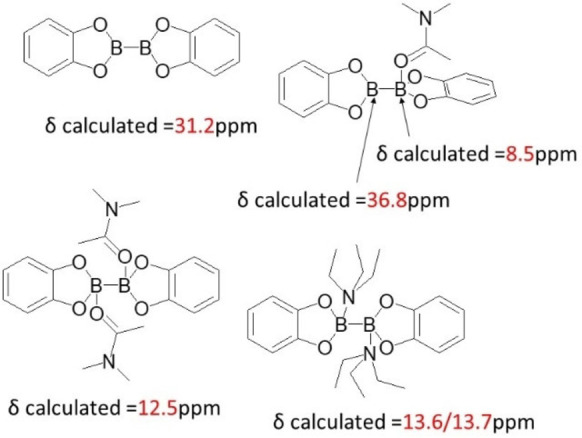
calculated ^11^B NMR by B3LYP‐D3BJ/pcSseg‐2.

We then calculated the chemical shift of ^11^B of likely possible boron complexes that may form and compared these results with the experimental results. From Figure 1, B_2_cat_2_ had its chemical shift at 31.2 ppm consistent with the literature reported value.^[36]^ One of the ^11^B peaks shifts from 25.4 ppm to 30 ppm in the 100 °C DMA environment.^[24]^ The B_2_cat_2_ can move faster and breaking the interaction with DMA solvent. Such chemical shift is equivalent to dichloromethane. This indicates 25.4 ppm refers to the boron that interact with DMA solvent, but it does not form boron oxygen bond and haves 2 empty p orbitals.

Next, we discuss the ^11^B NMR of the B_2_cat_2_ with two DMA complex with the two Et_3_N complex. Since the boron in the B_2_cat_2_ with two DMA and with two Et_3_N complexes are 4‐coordinate, the prediction of the chemical shifts in ^11^B would be around 13 ppm. The calculations are also consistent with the values reported in the literature.^[35]^

Finally, we will focus on the B_2_cat_2_ with one DMA complex. Our calculation results show that the predicted chemical shift would be 8.5 ppm for a 4‐coordinate center and 36.8 ppm for a 3‐coordinate center. Thes ^11^B peaks results are consistent with the trends when one sp^2^ and one sp^3^ center boron compounds formed in the literature.[^37^, ^38^] From the computed NMR data, it is suggested that the B_2_cat_2_ in DMA is less likely to be monosubstituted.

By the negative mode of ESI mass spectrum of B_2_cat_2_ in DMF^[39]^ (The spectrum can refer to Supporting information S58 of Gong's work), it show the detection of species is free B_2_cat_2_, [Bcat_2_]^−^ and B_2_cat_2_ with two DMF ligands. B_2_cat_2_ with one DMF ligand is not observed in the spectrum. This support our calculation the 13.8 ppm should be consider as B_2_cat_2_ with 2 DMF compunds. [Bcat_2_]^−^ cannot replace the role of borylation.(The experiment detailed can referred to Supporting information S29, 4.3.1 of Li's work)^[40]^ Li's work directly uses the NaBcat_2_ as control experiment and reported that the borylation failed. It indicates the reaction role of B_2_cat_2_ complex in DMA cannot be replaced by [Bcat_2_]^−^ .The remaining is B_2_cat_2_ with two DMF ligand as active intermediate.

**Figure 2:**
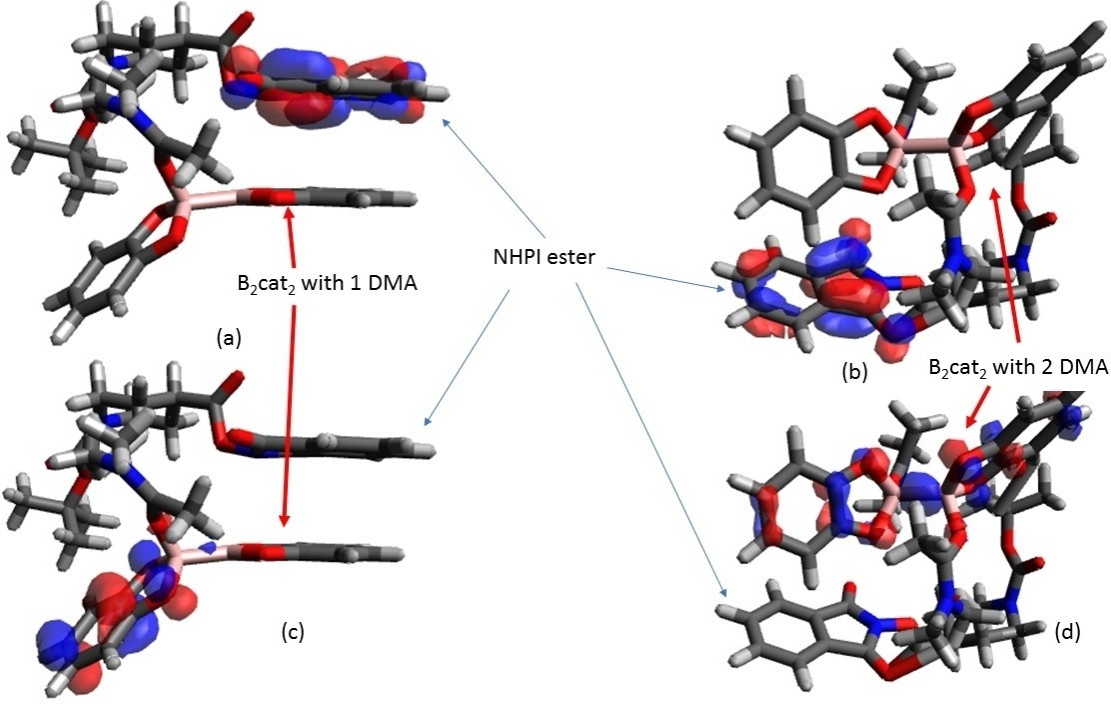
(a) LUMO of mixture of B_2_cat_2_ with 1 DMA and Boc‐NHPI ester. (b) LUMO of mixture of B_2_cat_2_ with 2 DMA and Boc‐NHPI ester. (c) HOMO of mixture of B_2_cat_2_ with 1 DMA and Boc‐NHPI ester (d) HOMO of mixture of B_2_cat_2_ with 2 DMA and Boc‐NHPI ester with isocontour value±0.05 is calculated by B3LYP/6‐31+G(d).

**Figure 3:**
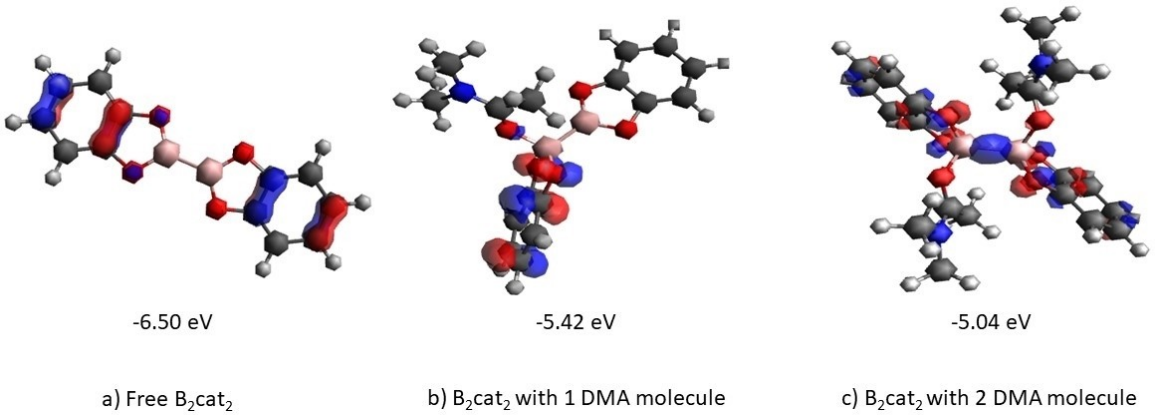
(a) HOMO of free B_2_cat_2_ (b) HOMO of B_2_cat_2_ with 1 DMA (c) HOMO of B_2_cat_2_ with 2 DMA isocontour value±0.06 is calculated by B3LYP/6‐31+G(d).

More theoretical evidence is needed to support the boron complex is indeed a complex of B_2_cat_2_ with 2 DMA. The TD‐DFT results of the mixture of tert‐Butyl carbamates protecting group with N‐hydroxyphthalimide(BOC‐NHPI) ester with two boron complexes are available in the Supporting information (S5–S8). This result shows that the transition from the highest occupied molecular orbital (HOMO) of boron complex to the π* of NHPI group. The orbital character of mixture is shown in Figure 2. Therefore, we then focus on the HOMO of the boron compound in Figure 3.

From Figure 3, the HOMO of B_2_cat_2_ has π character with an energy of −6.50 eV. The HOMO of B_2_cat_2_ with one DMA molecule is still of π character, but the energy rises to −5.42 eV. Finally, the HOMO of B_2_cat_2_ with two DMA molecules changes to sigma bond between boron and boron and its energy further rises to −5.04 eV. The EDA complex means charge transfer from the donor to the acceptor by excitation, The disubstituted B_2_cat_2_ can more easily donate an electron as it had a higher energy and a narrower energy gap for donating the electron to the NHPI ester. On the other side, B_2_cat_2_ with one DMA molecule required more energetic excitation for charge transfer.

**Figure 4:**
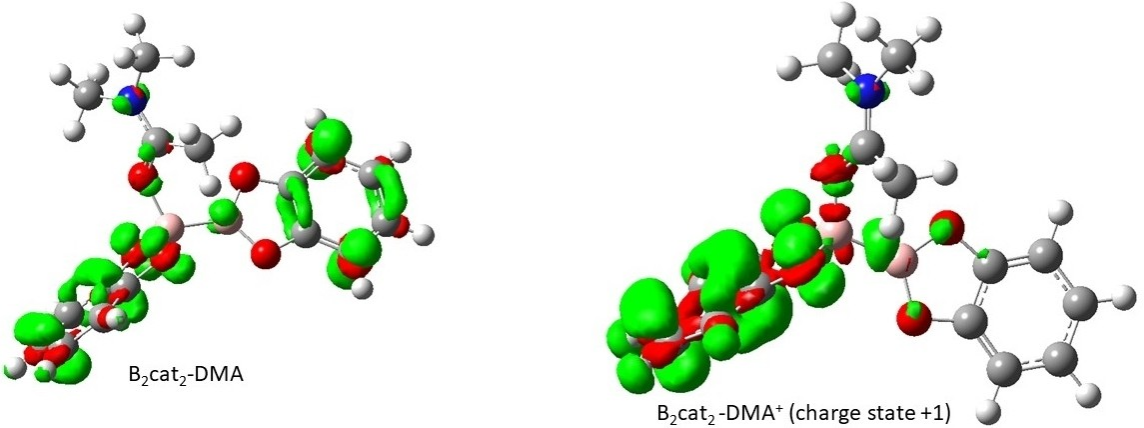
The plot of Fukui function (f^0^(r)) of B_2_cat_2_‐DMA (left panel) and B_2_cat_2_‐DMA^+^ (right panel) with isocontour value±0.004.

As the experiment determined that the reaction involved radical formation.^[15]^ Another way to look at this would be the Fukui function of B_2_cat_2_ with one DMA molecule. From Figure 4, the left panel represents neutral, and the right panel represents the cation. Then, both boron atoms in B_2_cat_2_ with one DMA complex do not have a large affinity to the radical reaction when compared to another region. This indicates that the radical attack on boron is not an easily available option in the proposed mechanism. The NBO spin density analysis of the cation of B_2_cat_2_ with 1 DMA molecule also supports boron will not attract any radical.

**Scheme 2:**
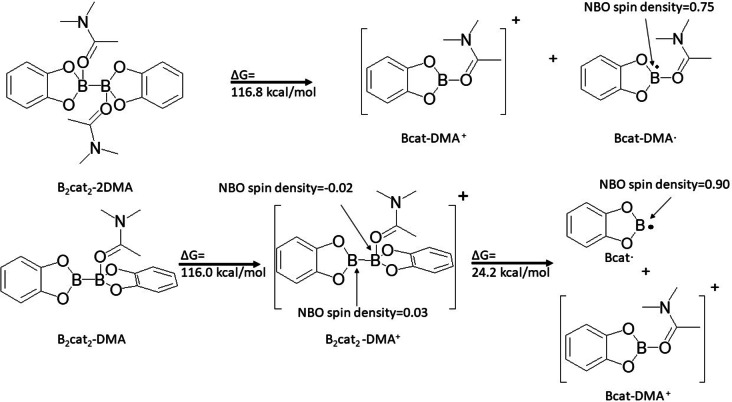
The ionization free energy of B_2_cat_2_ with 2 DMA (above) and with 1 DMA (below) by B3LYP‐D3BJ/6‐31+g(d**)**.

From Scheme 2, this shows the free energy of ionization. The free energy of B_2_cat_2_ with two DMA complex would be 116.8 kcal/mol. Since the HOMO of B_2_cat_2_ with two DMA complex is a boron‐boron sigma bond, breaking this sigma bond of the B_2_cat_2_ with two DMA complex would lead toward a cation complex (Bcat‐DMA^+^) and a radical complex (Bcat‐DMA ⋅ ). Then, the free energy of the B_2_cat_2_ with one DMA complex would be 116.0 kcal/mol. This structure can further fragment into one Bcat and one Bcat with one DMA molecule. Based on an NBO spin population analysis, the fragment of Bcat would be considered as a radical (Bcat ⋅ ),which has NBO spin density 0.9, and the fragment of Bcat with one DMA molecule complex would be considered as a cation.(Bcat‐DMA^+^) This process takes 24.2 kcal/mol energy. Since the Bcat radical is a very unstable species without any ligand attached and it also take more energy to generate, which also suggest the B_2_cat_2_ with two DMA is a better option for the reaction to proceed via this route. In short, B_2_cat_2_ with one DMA should not be considered in the reaction mechanism of C−B bond formation.

Therefore, we need a new perspective to interpret the ^11^B NMR of B_2_cat_2_ in DMA conditions. The proposed system with a value of 25.4 ppm could be considered as the free B_2_cat_2_ and the system with a value of 13.8 ppm for B_2_cat_2_ with 2 DMA molecules could be considered for the reaction system. Since the binding free energy for B_2_cat_2_ and DMA is positive, it exists in chemical equilibrium between the free B_2_cat_2_ and the B_2_cat_2_ with two DMA solvent molecules complex. The molar equivalent of the B_2_cat_2_ to DMA solvent molecule is one to a thousand. Then, it is easier to have 1 : 2 binding instead of 1 : 1 binding.

This assumption can be further supported by the experimental results of B_2_cat_2_ in DMA at 100 °C.^[24]^ They added 4,4’‐di‐tert‐butyl‐2,2’‐bipyridine at 100 °C into the solution mixture and recorded the ^11^B NMR after 1 hour and 4 hours. The chemical shift of 20 ppm was predominant after 1 hour condition. They calculated the ^11^B of B_2_cat_2_ with 4,4’‐di‐tert‐butyl‐2,2’‐bipyridine and this matches with the chemical shift. Then, the chemical shift of 14 ppm was predominant after the 4‐hour condition. This implies that 14 ppm and 25.4 ppm should be referred to different species.

Another ^11^B NMR experiment also came from the work of Aggarwal and coworkers.^[13]^ First, they measured B_2_cat_2_ in DMF condition. They reported two chemical shifts, 13.8 ppm and 23.1 ppm. Then, they added the 2‐iodophenyl‐thionocarbonate to the solution and reported no change in the chemical shift. They also measured the ^11^B NMR of B_2_cat_2_ with Et_3_N in DMF conditions. We noticed their reported spectrum had two obvious changes. An additional 7.5 ppm chemical shift can be recorded, and the intensity of the original chemical shift changed. As previously mentioned, the 7.5 ppm should be assigned as the sp^3^ boron in monosubstituted B_2_cat_2_. Since the interaction of DMF and the vacant orbital of the sp^2^ boron, we think that the signal would be merged to free B_2_cat_2_. Then, it was obvious that the intensity of chemical shift at 23.1 ppm decreased while that at 13.8 ppm increased. The 23.1 ppm originally came from the free B_2_cat_2_. Since the addition of Et_3_N would also bind to free B_2_cat_2_, that can explain why the intensity of 13.8 ppm increased. In the DFT optimization, there was a different bond length between the boron and the nitrogen bond and hence this predicts two chemical shifts for boron. Since the variation is not large, we still assign the 13.8 ppm chemical shift can come from B_2_cat_2_ with 2 DMA and B_2_cat_2_ with 2 Et_3_N.

To sum up, we propose that the B_2_cat_2_ with 2 DMA complex and the B_2_cat_2_ with 2 Et_3_N complex for 2‐iodophenyl‐thionocarbonate reaction could be considered as donor reagents.

**Scheme 3:**
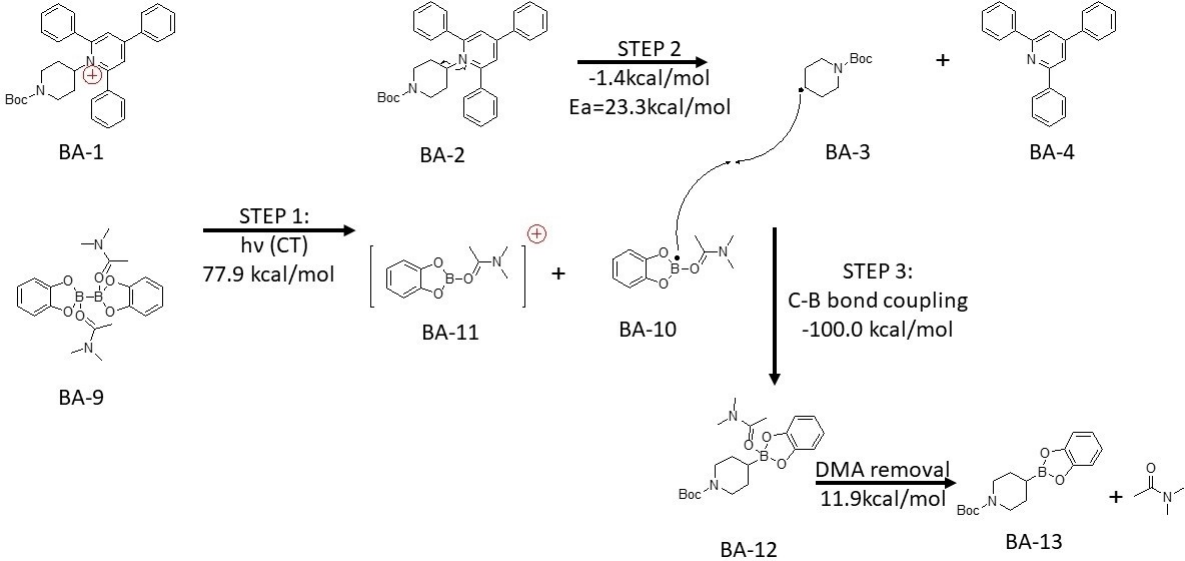
The proposed mechanism for Katritzky salt and N‐hydroxyphthalimide ester with B_2_cat_2_ in DMA. Electronic energy of elementary step reaction by DSDPBEP‐86 with 6‐31+g(d) in DMA (SMD solvation model) Ea stand for activation energy.

**Scheme 4:**
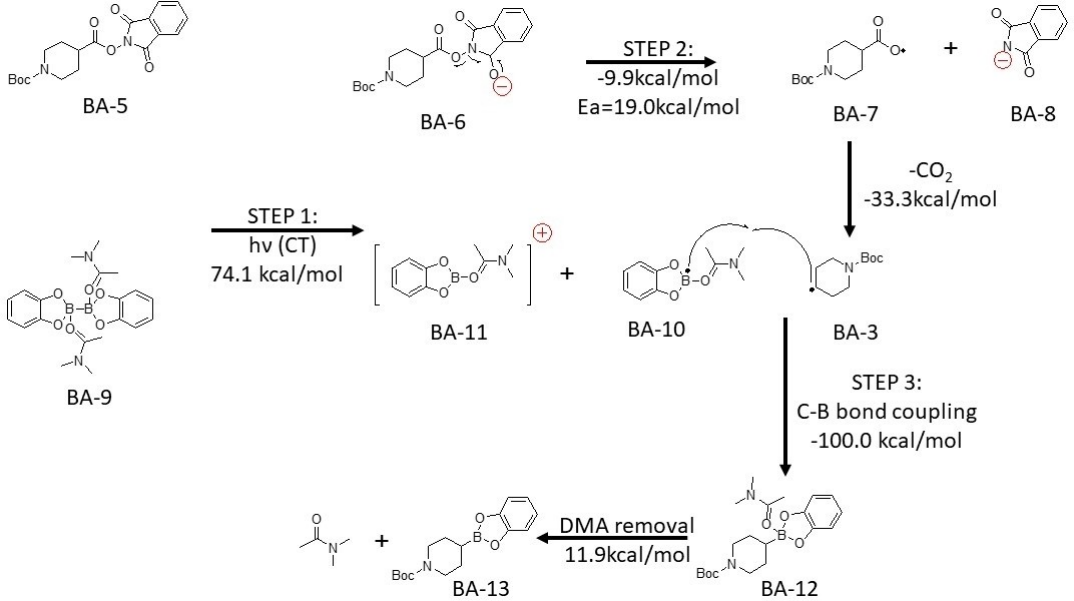
The proposed mechanism for N‐hydroxyphthalimide ester with B_2_cat_2_ in DMA. Electronic energy of elementary step reaction by DSDPBEP‐86 with 6‐31+g(d) in DMA (SMD solvation model). Ea stand for activation energy.

B_2_cat_2_ in dichloromethane failed to undergo charge transfer. This means the free B_2_cat_2_ cannot act as the donor. Therefore, we can only have disubstituted B_2_cat_2_ that could likely be the species in the proposed reaction mechanism. In Scheme 3 and Scheme 4, the first step would be light activated charge transfer from the disubstituted B_2_cat_2_ to the Katritzky salt or the N‐hydroxyphthalimide ester. (BA-1+BA-9→BA-2+BA-10+BA-11 for Katritzky salt; BA-5+BA-9→BA-6+BA-10+BA-11 for NHPI ester) Then, the Katritzky salt or N‐hydroxyphthalimide ester would undergo fragmentation to form a carbon radical. (BA-2→BA-3+BA-4 for Katritzky salt; $BA6\to BA-7+BA-8\to BA-3+CO_{2}$ for NHPI ester)Finally, the coupling between carbon and boron would take place in the proposed mechanism. (BA-3+BA-10→BA-12→BA-13+DMA) .

**Scheme 5:**
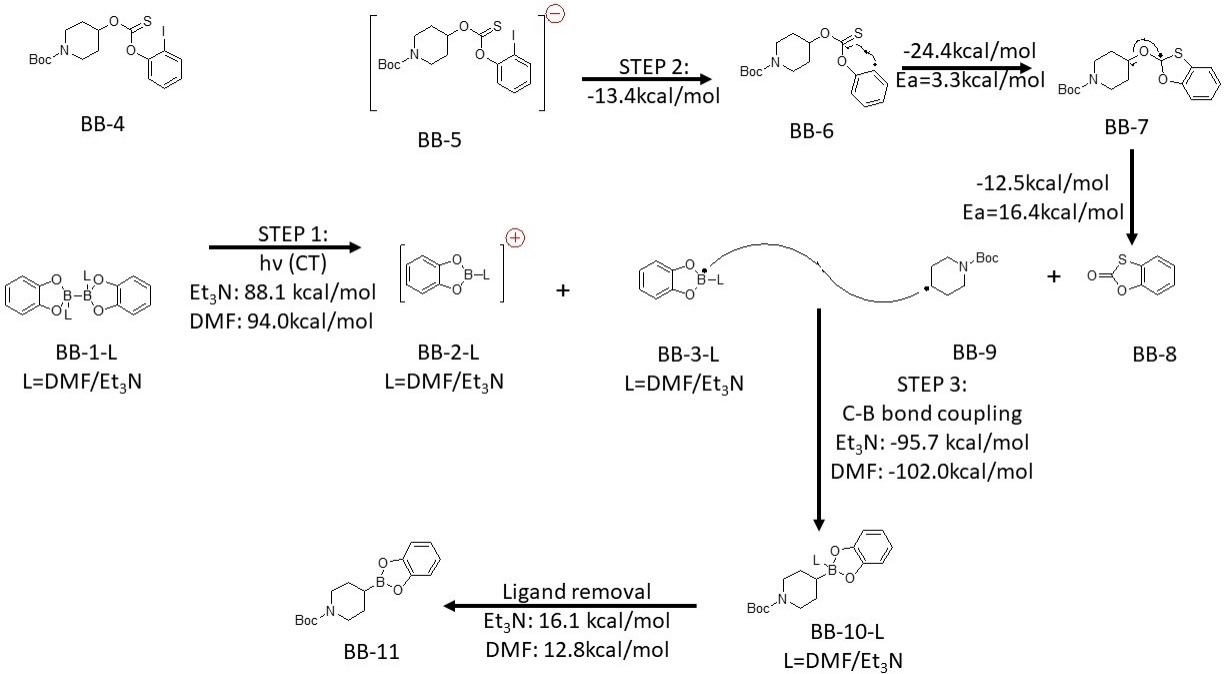
The proposed mechanism for 2‐iodophenyl‐thionocarbonates with B_2_cat_2_ in DMF Electronic energy of elementary step reaction by DSDPBEP‐86 with 6‐31+g(d) in DMA (SMD solvation model). Ea stand for activation energy.

For 2‐iodophenyl‐thionocarbonates, the reaction mechanism resembles the previous one dsicussed. The difference would be the addition of the disubstituted B_2_cat_2_ with Et_3_N also acts as a donor in this condition. In Scheme 5, the first step would be light activated charge transfer from DMF or Et_3_N disubstituted B_2_cat_2_ to the 2‐iodophenyl‐thionocarbonates. (BB-1-L+BB-4→BB-2-L+BB-3-L+BB-5 ). Then, the 2‐iodophenyl‐thionocarbonates would fragment to form a radical. ($BB-5\to BB-6+I^{-}\to BB-7\to BB-8+\;BB-9$ ). Finally, the coupling between the carbon and boron also takes place. (BB-3-L+BB-9→BB-10-L→BB-11 ).

Finally, we consider the reaction mechanism of the aryl N‐hydroxyphthalimide ester with the B_2_pin_2_ conditions. Glorius and co‐workers reported their reaction would not proceed via the EDA complex due to no additional absorption peak can be recorded in the UV‐Vis spectrum.^[23]^ Then, they further perform a Stern‐Volmer fluorescence analysis to study the reaction mechanism. The reaction mechanism and further proposed that the aryl N‐hydroxyphthalimide ester is excited and relaxed to a triplet state that is then followed by quenching with the B_2_pin_2_ pyridine complex.

Based on the observation of Pietsch's work, the neutral Lewis base cannot form an adduct with B_2_pin_2_.^[37]^ Such experimental observations affect the reaction mechanism considerations. The aryl N‐hydroxyphthalimide ester is viewed as an electron acceptor. Then, it looks for the electron donor. Pyridine is classified as an electron acceptor.^[18]^ Therefore, it seems the remaining B_2_pin_2_ act as an electron donor for the complete reaction.

**Scheme 6:**
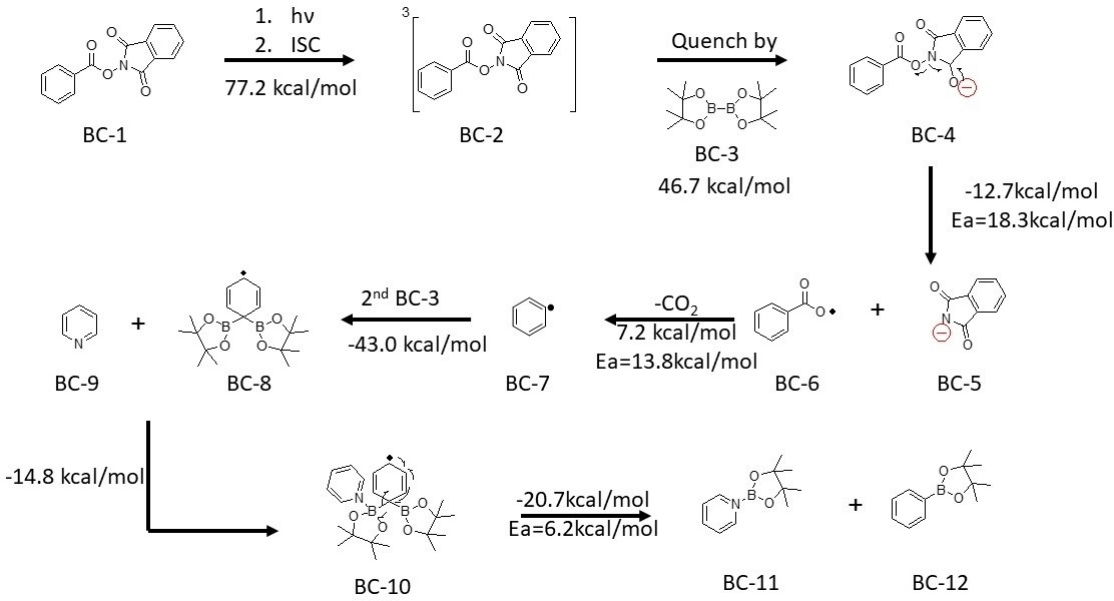
The reaction mechanism for aryl N‐hydroxyphthalimide Esters. Electronic energy of elementary step reaction by DSDPBEP‐86 with 6‐31+g(d) in ethyl acetate (SMD solvation model) Ea stand for activation energy.

In Scheme 6, summarizes our proposed reaction mechanism. The first step would be the aryl N‐hydroxyphthalimide ester is excited and intersystem crossing to the triplet state. (BC-1→BC-2 ). Then, it would be quenched by B_2_pin_2_ (BC-2+BC-3→BC-4 ), followed by fragmentation to generate a carbon radical. ($BC-4\to BC-5+BC-6\to BC-7+CO_{2}$ ). We performed a Fukui function analysis of the B_2_pin_2_ cation radical. These results show that the boron in the B_2_pin_2_ cation radical does not prefer the radical attack. Then, we further plot the Fukui function of B_2_pin_2_ (neutral form), these results show a radical affinity located between the boron and other boron. The two plots are available in the Supporting Information. (S18–S19) Therefore, the carbon radical would be going to bind with the second B_2_pin_2_ molecule. (BC-7+BC-3→BC-8 ) Then, the pyridine would come to attack one of the boron atoms (BC-8+BC-9→BC-10 ), followed by boron carbon bond cleavage to yield the product. (BC-10→BC-11+BC-12) .

When looking at the energy for the charge transfer (D‐A to D^+^‐A^−^) state, the non‐substituted B_2_pin_2_ takes more electronic energy than the substituted B_2_cat_2_. The total energy for B_2_pin_2_ and aryl N‐hydroxyphthalimide esters require 123.9 kcal/mol. While the substituted B_2_cat_2_ and aliphatic N‐hydroxyphthalimide esters require only 74.1 kcal/mol. This indicates the ligand substituent to the donor will lower the energy of the charge transfer. This also explains why 35 °C is preferred for B_2_pin_2_ and aryl N‐hydroxyphthalimide esters. This required approximately an extra 50 kcal/mol for the charge transfer. When compared with different functional groups, the Katritzky salt takes 77.9 kcal/mol and resembles that for the N‐hydroxyphthalimide esters. While 2‐iodophenyl‐thionocarbonates required 94.0 kcal/mol and approximately an extra 20 kcal/mol for the charge transfer. Therefore, Aggarwal and coworkers added Et_3_N to the mixture and this would reduce the energy to 88.1 kcal/mol for the charge transfer.

For fragmentation of an anion and the generation of a carbon radical, the Katritzky salt has the least exothermic process and only releases −1.4 kcal/mol. Then, the aliphatic NHPI ester released −43.2 kcal/mol of energy. Finally, the anion of the 2‐iodophenyl‐thionocarbonates is the most exothermic of the reactions studied in this work and releases −50.3 kcal/mol.

For carbon‐boron bond formation and the yield of the products, the B_2_cat_2_ and B_2_pin_2_ source is found to be exothermic by around −88 kcal/mol and −78.5 kcal/mol respectively.

The computations found that for the overall reactions, the aryl NHPI ester and B_2_pin_2_ is endothermic by about 39.9 kcal/mol to occur while the aliphatic NHPI ester and B_2_cat_2_ reaction take has about −57.2 kcal/mol of energy released This indicates the substitution of the ligand can change the reaction from endothermic to exothermic. The Katritzky salt is exothermic by about −11.6 kcal/mol. Finally, the 2‐iodophenyl‐thionocarbonates reaction with the B_2_cat_2_ and Et_3_N complex was about −41.8 kcal/mol and B_2_cat_2_ and DMF complex is exothermic by −45.5 kcal/mol.

## Conclusions

In this journal, we have reviewed the photoinduced borylation reaction form Aggarwal and Glorius work.

We determine the ratio of the bis(catecholato)diboron with DMA complex should be a 1 : 2 complex, instead of 1 : 1 ratio. After know the 1 : 2 complex is involved in the reactions of interest, we propose reaction mechanisms based on charge transfer from the B_2_cat_2_ complex to different acceptor groups. The acceptor would then undergo fragmentation to form a radical species that then undergoes radical coupling.

We also worked out the overall reaction mechanism for the photoinduced borylation via a source of B_2_pin_2._for the reactions of interest in this study.

We also compare the energy of different boron source, B_2_cat_2_ and B_2_pin_2_. The main difference of B_2_cat_2_ can form adduct with neutral Lewis base, while B_2_pin_2_ can only form adduct with anionic ligand. Such variation affect the reaction mechanism and energy of each steps. In our calculation, the NHPI ester is the best among three leaving group.

## Supporting Information

The authors have included the NMR of GIAO data and optimized coordinates of the structures involved in this study in the Supporting Information.

## Conflict of interests

The authors declare no conflict of interest.

1

## Supporting information

As a service to our authors and readers, this journal provides supporting information supplied by the authors. Such materials are peer reviewed and may be re‐organized for online delivery, but are not copy‐edited or typeset. Technical support issues arising from supporting information (other than missing files) should be addressed to the authors.

Supporting Information

## Data Availability

The data that support the findings of this study are available in the supplementary material of this article.
