# Supplementary material for: A Computational Study of Photoinduced Borylation for Selected Boron Sources
Source: ChemistryOpen. 2024 Mar 8;13(7):e202300285. doi: 10.1002/open.202300285 (PMC11230929; doi:10.1002/open.202300285)
Supplement: Supplementary file 1 — Supporting Information [file OPEN-13-e202300285-s001.pdf]

ChemistryOpen

Supporting Information

A Computational Study of Photoinduced Borylation for Selected Boron Sources

Ka Wa Fan, Hoi Ling Luk, and David Lee Phillips*

Supporting information

Contents

NMR of GIAO data....4

TD-DFT of Boc-NHPI ester with B2cat2˙1DMA complex....6

TD-DFT of Boc-NHPI ester with B2cat2˙2DMA complex....7

XYZ coordinate of B2cat2˙2DMA....10

XYZ coordinate of B2cat2˙1DMA....11

XYZ coordinate of Bcat˙DMA positive charge....12

XYZ coordinate of Bcat˙DMA rad (spin located on Boron)....13

XYZ coordinate of Bcat˙DMA rad (spin located on carbon)....14

XYZ coordinate of B2cat2˙1DMA positive charge....15

XYZ coordinate of fragmented B2cat2˙1DMA positive charge....16

Lewis's structure of Bcat with 1 DMA solvent molecules....18

The Fukui function of B_2pin_2....19

XYZ coordinate of BA-1....21

XYZ coordinate of BA-2....23

XYZ coordinate of BA-3....25

XYZ coordinate of BA-4....26

XYZ coordinate of product of BA-2....27

XYZ coordinate of transition state BA-2....29

XYZ coordinate of BA-5....31

XYZ coordinate of BA-6....32

XYZ coordinate of product of BA-6....33

XYZ coordinate of transition state of BA-6....35

XYZ coordinate of BA-7....36

XYZ coordinate of product of BA-7....37

XYZ coordinate of BA-8....38

XYZ coordinate of BA-9....39

XYZ coordinate of BA-10 40
XYZ coordinate of BA-11 41
XYZ coordinate of BA-12 42
XYZ coordinate of BA-13 44
XYZ coordinate of BB-1-DMF..45
XYZ coordinate of BB-2-DMF..46
XYZ coordinate of BB-3-DMF..47
XYZ coordinate of BB-1-Et_3N 48
XYZ coordinate of BB-2-Et_3N 50
XYZ coordinate of BB-3-Et_3N 51
XYZ coordinate of BB-4 52
XYZ coordinate of BB-5 53
XYZ coordinate of product of BB-5..55
XYZ coordinate of BB-6 56
XYZ coordinate of transition state of BB-6 57
XYZ coordinate of BB-7 58
XYZ coordinate of transition state of BB-7 60
XYZ coordinate of product of BB-7..61
XYZ coordinate of BB-8 62
XYZ coordinate of BB-9 63
XYZ coordinate of BB-10-DMF..64
XYZ coordinate of BB-10-Et_3N 65
XYZ coordinate of BB-11 67
XYZ coordinate of BC-1..68
XYZ coordinate of BC-2..69
XYZ coordinate of BC-3..70
XYZ coordinate of BC-4..71
XYZ coordinate of product of BC-4..72
XYZ coordinate of transition state of BC-4 73
XYZ coordinate of BC-5..74
XYZ coordinate of BC-6..74
XYZ coordinate of transition state of BC-6 75
XYZ coordinate of BC-7..75
XYZ coordinate of BC-8..76
XYZ coordinate of BC-10..77
XYZ coordinate of transition state of BC-10 79
XYZ coordinate of BC-11+BC-12 81

NMR of GIAO data

B3LYP-D3BJ pcsSeg-2

BF3OEt2

```
     1  B    Isotropic =    97.4151   Anisotropy =     7.1120
   XX=    91.5634   YX=     4.9601   ZX=    -1.8878
   XY=     4.5698   YY=    99.8652   ZY=     1.0723
   XZ=    -2.1213   YZ=     1.4847   ZZ=   100.8167
   Eigenvalues:    88.9285   101.1604   102.1564
```

B2cat2

```
  11  B    Isotropic =    66.2474   Anisotropy =    25.7903
   XX=    68.4936   YX=     0.0003   ZX=    -0.0007
   XY=    -0.0000   YY=    46.8850   ZY=    -1.4424
   XZ=    -0.0083   YZ=    -1.9172   ZZ=    83.3638
   Eigenvalues:    46.8078    68.4936    83.4410
    22  B    Isotropic =    66.2474   Anisotropy =    25.7903
   XX=    68.4936   YX=    -0.0003   ZX=     0.0004
   XY=     0.0000   YY=    46.8850   ZY=     1.4423
   XZ=    -0.0065   YZ=     1.9171   ZZ=    83.3638
   Eigenvalues:    46.8078    68.4936    83.4410
```

B2cat2˙1DMA

```
   11  B    Isotropic =    88.8724   Anisotropy =     9.1638
   XX=    91.7855   YX=     2.5446   ZX=    -0.0050
   XY=     7.7459   YY=    86.6898   ZY=    -1.3127
   XZ=     0.4831   YZ=     0.0564   ZZ=    88.1420
   Eigenvalues:    83.4042    88.2315    94.9816
   22  B    Isotropic =    60.5808   Anisotropy =    36.9305
   XX=    73.6373   YX=    -6.7545   ZX=     4.8347
   XY=    -6.6245   YY=    38.5630   ZY=    26.6599
   XZ=     4.5070   YZ=    27.2837   ZZ=    69.5421
   Eigenvalues:    21.6773    74.8639    85.2012
```

B2cat2˙2DMA

```
   11  B    Isotropic =    84.8956   Anisotropy =    17.3813
```

```
XX=    93.5818   YX=     8.3156   ZX=     0.2983
XY=     3.5786   YY=    83.7298   ZY=    -0.1324
XZ=     2.0984   YZ=     1.6954   ZZ=    77.3752
Eigenvalues:    77.2596    80.9441    96.4831
 22  B    Isotropic =    84.8956   Anisotropy =    17.3818
XX=    93.5824   YX=     8.3157   ZX=    -0.2979
XY=     3.5784   YY=    83.7293   ZY=     0.1322
XZ=    -2.0978   YZ=    -1.6954   ZZ=    77.3751
Eigenvalues:    77.2595    80.9438    96.4834
```

B2cat2˙2Et3N

```
11  B    Isotropic =    83.6790   Anisotropy =    12.8749
XX=    91.5091   YX=    -0.4969   ZX=     3.2169
XY=     1.6111   YY=    80.3698   ZY=     2.7724
XZ=     2.7422   YZ=     0.5964   ZZ=    79.1581
Eigenvalues:    77.6208    81.1540    92.2623
22  B    Isotropic =    83.7526   Anisotropy =    14.0779
XX=    91.4718   YX=    -2.9359   ZX=    -5.1459
XY=     0.4494   YY=    80.9682   ZY=    -2.1181
XZ=    -4.4377   YZ=    -0.1131   ZZ=    78.8177
Eigenvalues:    76.7073    81.4126    93.1379
```

TD-DFT of Boc-NHPI ester with B2cat2·1DMA complex

Excited State 1: Singlet-A 2.4245 eV 511.38 nm f=0.0004
<S**2>=0.000
184 -> 185 0.70643
This state for optimization and/or second-order correction.
Total Energy, E(TD-HF/TD-DFT) = -2399.11722520
Copying the excited state density for this state as the 1-particle RhoCI density.

Excited State 2: Singlet-A 3.1197 eV 397.43 nm f=0.0082
<S**2>=0.000
183 -> 185 0.70291

Excited State 3: Singlet-A 3.5035 eV 353.88 nm f=0.0001
<S**2>=0.000
181 -> 185 0.11375
182 -> 185 0.68981

Excited State 4: Singlet-A 3.5097 eV 353.26 nm f=0.0000
<S**2>=0.000
181 -> 185 0.69563
182 -> 185 -0.11363

Excited State 5: Singlet-A 3.5858 eV 345.76 nm f=0.0013
<S**2>=0.000
180 -> 185 0.70145

Excited State 6: Singlet-A 3.7198 eV 333.31 nm f=0.0001
<S**2>=0.000
184 -> 186 0.70606

Excited State 7: Singlet-A 3.9338 eV 315.18 nm f=0.0003
<S**2>=0.000
175 -> 185 0.68437

Excited State 8: Singlet-A 3.9974 eV 310.16 nm f=0.0037
<S**2>=0.000
171 -> 185 0.10231

172 -> 185 0.10245
177 -> 185 0.67328

Excited State 9: Singlet-A 4.1877 eV 296.07 nm f=0.0330
<S**2>=0.000
184 -> 187 -0.25582
184 -> 188 0.55079
184 -> 189 -0.34983

Excited State 10: Singlet-A 4.2169 eV 294.02 nm f=0.0006
<S**2>=0.000
178 -> 185 0.69675

TD-DFT of Boc-NHPI ester with B2cat2˙2DMA complex

Excited State 1: Singlet-A 1.9101 eV 649.09 nm f=0.0002
<S**2>=0.000
207 -> 209 -0.16099
208 -> 209 0.68813
This state for optimization and/or second-order correction.
Total Energy, E(TD-HF/TD-DFT) = -2687.01981493
Copying the excited state density for this state as the 1-particle RhoCI density.

Excited State 2: Singlet-A 2.3105 eV 536.62 nm f=0.0002
<S**2>=0.000
207 -> 209 0.68751
208 -> 209 0.16114

Excited State 3: Singlet-A 3.1441 eV 394.34 nm f=0.0015
<S**2>=0.000
205 -> 209 0.46340
206 -> 209 0.53260

Excited State 4: Singlet-A 3.2563 eV 380.75 nm f=0.0006
<S**2>=0.000
207 -> 210 -0.18884

208 -> 210 0.68097

Excited State 5: Singlet-A 3.3870 eV 366.06 nm f=0.0000
<S**2>=0.000
205 -> 209 0.53293
206 -> 209 -0.46368

Excited State 6: Singlet-A 3.4747 eV 356.82 nm f=0.0001
<S**2>=0.000
203 -> 209 0.65586
204 -> 209 0.25913

Excited State 7: Singlet-A 3.5042 eV 353.82 nm f=0.0001
<S**2>=0.000
203 -> 209 -0.25917
204 -> 209 0.65509

Excited State 8: Singlet-A 3.6621 eV 338.56 nm f=0.0001
<S**2>=0.000
207 -> 210 0.68013
208 -> 210 0.18921

Excited State 9: Singlet-A 3.9223 eV 316.10 nm f=0.0001
<S**2>=0.000
197 -> 209 0.67863
201 -> 209 0.14973

Excited State 10: Singlet-A 3.9544 eV 313.53 nm f=0.0340
<S**2>=0.000
208 -> 212 0.69749

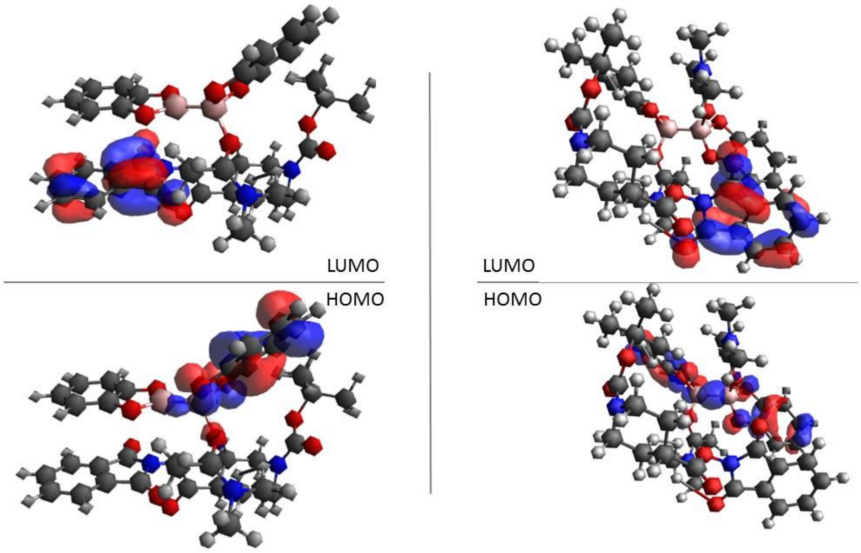

The orbital transition between the Boc-NHPI ester with the B_2cat_2 complex. The left panel represents monosubstituted and the right panel represents disubstituted. Two TD calculations show the transition would be from HOMO to LUMO. In both situations, the LUMO localized on the NHPI functional group. So that it is more important to look at the HOMO. The HOMO of monosubstituted shows pi the character and HOMO of disubstituted show the sigma boron-boron bond.

Also, the charge transfer process would help to break down the sigma boron-boron bond which can be coupled with the carbon radical.

XYZ coordinate of B2cat2˙2DMA

B3LYP-D3BJ/6-31+g(d)/SMD-DMA

C	-2.87053700	-0.84481400	0.02609900
C	-2.44970600	-1.28668400	-1.24512400
C	-3.35693100	-1.79517000	-2.16450000
C	-4.71563800	-1.85616700	-1.78741700
C	-5.13229700	-1.41613800	-0.52839900
C	-4.20517600	-0.89939800	0.40194900
H	-3.02798100	-2.13155000	-3.14457100
H	-5.44457100	-2.25169100	-2.49045400
H	-6.18375700	-1.46994600	-0.25725600
H	-4.52269800	-0.55070100	1.38148600
B	-0.58885500	-0.63744600	-0.07110600
C	2.87050800	0.84478800	0.02625500
C	2.44976800	1.28658300	-1.24502400
C	4.20512100	0.89938700	0.40219600
C	3.35706100	1.79500600	-2.16436900
C	5.13231000	1.41606500	-0.52811900
H	4.52257300	0.55074800	1.38177500
C	4.71574300	1.85601700	-1.78719400
H	3.02818200	2.13132500	-3.14448400
H	6.18375200	1.46988400	-0.25690600
H	5.44472700	2.25149400	-2.49020400
B	0.58883100	0.63741300	-0.07110300
O	-1.11654900	-1.10124600	-1.39556500
O	-1.82000500	-0.35978500	0.73017000
O	1.81992600	0.35979800	0.73027800
O	1.11662200	1.10114500	-1.39554800
O	0.01881500	-1.90963900	0.65004000
C	1.15226100	-2.44730200	0.38274400
N	1.71461600	-3.19539000	1.32364000
C	1.11230600	-3.30162200	2.65427800
H	0.44475800	-4.17004900	2.70195900
H	1.91547200	-3.42762900	3.38434200
H	0.54767100	-2.39860200	2.88208200
C	2.93501100	-3.97618300	1.11902000
H	2.78478000	-4.97133000	1.54790100

H	3.16200800	-4.08394100	0.06055200
H	3.77696600	-3.49282600	1.62702500
C	1.81266300	-2.29064000	-0.95579200
H	2.86552400	-2.01794800	-0.84870100
H	1.75807800	-3.23765500	-1.50654700
H	1.29893600	-1.53033200	-1.53892500
O	-0.01888700	1.90964100	0.64993200
C	-1.15227500	2.44734600	0.38247900
N	-1.71466400	3.19553100	1.32328000
C	-1.81257400	2.29060200	-0.95610300
C	-1.11243900	3.30183000	2.65394800
C	-2.93501700	3.97636400	1.11854300
H	-2.86543800	2.01789600	-0.84907100
H	-1.75796700	3.23758500	-1.50690800
H	-1.29879800	1.53026600	-1.53915600
H	-0.44501000	4.17034600	2.70166400
H	-1.91565600	3.42772800	3.38397600
H	-0.54769600	2.39888400	2.88177200
H	-2.78479500	4.97150100	1.54745100
H	-3.16190400	4.08414700	0.06005500
H	-3.77704000	3.49303100	1.62645700

XYZ coordinate of B2cat2˙1DMA

B3LYP-D3BJ/6-31+g(d)/SMD-DMA

C	-2.50780200	-1.41430400	-0.75432300
C	-2.54688900	-1.26540800	0.64486100
C	-3.62931100	-1.71126000	1.38823300
C	-4.69302700	-2.32528300	0.69436700
C	-4.65399200	-2.47495300	-0.69449100
C	-3.54987700	-2.01691300	-1.44350800
H	-3.65234700	-1.59271900	2.46834400
H	-5.55379000	-2.68446300	1.25268900
H	-5.48435900	-2.95053500	-1.21020800
H	-3.51331600	-2.13126300	-2.52368200
B	-0.66348000	-0.24235000	-0.12167900
C	3.02668400	-1.41830700	0.25962500
C	3.21595500	-0.22887600	-0.44549300

C	4.08704600	-2.22833100	0.63273900
C	4.47490900	0.21461500	-0.81635100
C	5.37063900	-1.79190100	0.26331300
H	3.93216800	-3.15263400	1.18116700
C	5.56052100	-0.59679100	-0.44531100
H	4.61387800	1.14125100	-1.36503900
H	6.23251100	-2.39547700	0.53384200
H	6.56704500	-0.28936200	-0.71492900
B	1.02974500	-0.49230300	-0.09080900
O	-1.40690700	-0.67120500	1.09091900
O	-1.34358700	-0.91877400	-1.25318100
O	1.67843100	-1.59456800	0.48616300
O	1.98762000	0.35560000	-0.67156900
O	-0.98080600	1.27894000	-0.36649600
C	-0.63957900	2.27630700	0.36971900
N	-0.92511000	3.49135800	-0.07108900
C	-1.65927900	3.69758000	-1.32289000
H	-2.37160700	4.51267200	-1.17247200
H	-0.96150000	3.97257700	-2.12162800
H	-2.19227500	2.79019600	-1.59992900
C	-0.54417400	4.70929900	0.65044400
H	-1.35739300	5.02978600	1.31120500
H	0.36415900	4.55373200	1.23047100
H	-0.35478900	5.49183600	-0.08709700
C	0.05473800	2.10011600	1.68910200
H	-0.40668100	2.72967800	2.45520900
H	-0.00251900	1.06323500	2.01363500
H	1.10786600	2.39177900	1.60156600

XYZ coordinate of Bcat·DMA positive charge

Identical to BA-11

B3LYP-D3BJ/6-31+g(d)/SMD-DMA

C	-2.15971800	-0.73643400	0.21973100
C	-1.94756400	0.56019900	-0.24957100
C	-2.98786400	1.40648100	-0.58347900
C	-4.28330800	0.88595700	-0.42382300

C	-4.49755300	-0.41647900	0.04766400
C	-3.42745600	-1.26318600	0.38266800
H	-2.81257000	2.41350700	-0.94817100
H	-5.13590100	1.51063300	-0.67330200
H	-5.51355900	-0.78381900	0.15801300
H	-3.58438700	-2.27280100	0.74865600
B	-0.00774100	-0.37087700	0.13923400
O	-0.57604500	0.80222400	-0.30792900
O	-0.93029500	-1.33797800	0.47021400
O	1.34409800	-0.63980500	0.20337800
C	2.34742200	0.23295600	0.28873400
N	3.49112900	-0.16307500	-0.18159200
C	3.69846000	-1.49437800	-0.77447700
H	3.77377000	-1.38369700	-1.86007800
H	4.63817100	-1.88719100	-0.38129500
H	2.88011200	-2.16285700	-0.51980200
C	4.68348900	0.70160000	-0.14691600
H	5.30749700	0.43057200	-0.99900100
H	4.40319000	1.75021900	-0.22610100
H	5.23183800	0.52029800	0.78239700
C	2.14444300	1.55422000	0.94637100
H	2.99071600	1.78884900	1.59633600
H	2.06569100	2.34158000	0.18642900
H	1.23206900	1.54947100	1.54417900

XYZ coordinate of Bcat˙DMA rad (spin located on Boron)

B3LYP-D3BJ/6-31+g(d)/SMD-DMA

C	-2.09705200	0.70194600	-0.33436900
C	-1.92939600	-0.65232900	0.00315100
C	-2.96285800	-1.39522500	0.55413600
C	-4.19329200	-0.74025100	0.76540600
C	-4.36062400	0.60655600	0.42917600
C	-3.30466900	1.35362000	-0.13138200
H	-2.82545700	-2.44114000	0.81452000
H	-5.02283600	-1.29593000	1.19484500
H	-5.31945200	1.08951000	0.59896100

H	-3.42817600	2.40099800	-0.39285400
B	-0.01146200	0.08636700	-0.93161500
O	-0.66236200	-1.05996300	-0.29946200
O	-0.94439300	1.20234500	-0.86460400
O	1.27844300	0.46526000	-0.33327800
C	2.33320600	-0.27987800	-0.19727300
N	3.41546100	0.28867700	0.31244200
C	3.44558500	1.71202000	0.66024500
H	3.27162800	1.83641400	1.73547500
H	4.43430400	2.10334500	0.40881600
H	2.68551600	2.25492500	0.10200400
C	4.62985800	-0.47216300	0.62428200
H	5.11262900	0.00552300	1.47922900
H	4.38617900	-1.49914900	0.89522800
H	5.31931900	-0.46331400	-0.22695000
C	2.32057400	-1.69387700	-0.66842300
H	3.31751400	-2.05661600	-0.92084000
H	1.87920800	-2.35145200	0.08845900
H	1.67720600	-1.73935200	-1.55686500

XYZ coordinate of Bcat˙DMA rad (spin located on carbon)

B3LYP-D3BJ/6-31+g(d)/SMD-DMA

0 2

C	2.13501900	-0.76357700	-0.13167800
C	2.00395100	0.61488400	0.06145200
C	3.09389700	1.43189500	0.30181200
C	4.35247600	0.80524300	0.34810200
C	4.48500900	-0.57645800	0.16192400
C	3.36675200	-1.39325800	-0.08417800
H	2.98134000	2.50233800	0.44408400
H	5.23675600	1.40872900	0.53258900
H	5.47089900	-1.03080100	0.20486900
H	3.46340800	-2.46408500	-0.23306800
B	0.00703500	-0.22621300	-0.36258500
O	0.67277500	0.96539600	-0.04225800
O	0.89426300	-1.31393800	-0.36543500

O	-1.31988000	-0.39975600	-0.52903800
C	-2.35068500	0.39379100	0.02208200
N	-3.55239700	-0.31268100	-0.02666200
C	-3.53973000	-1.68265700	0.48165800
H	-3.53015900	-1.70456200	1.58534600
H	-4.43695900	-2.19869600	0.12851100
H	-2.66215800	-2.21220000	0.11137600
C	-4.76645700	0.41301800	0.32455900
H	-4.79100600	0.68952400	1.39290900
H	-4.86716100	1.31695000	-0.27763700
H	-5.62451900	-0.23040700	0.11505100
C	-2.34332300	1.84711900	-0.34966500
H	-2.96427700	2.42957100	0.33750600
H	-1.32751300	2.24220900	-0.29118500
H	-2.71576200	2.01764500	-1.37335000

XYZ coordinate of B2cat2˙1DMA positive charge

B3LYP-D3BJ/6-31+g(d)/SMD-DMA

C	-2.45295100	-1.30448800	-0.78455800
C	-2.49898900	-1.20686900	0.65597200
C	-3.42368300	-1.94308700	1.40924100
C	-4.28382100	-2.76120700	0.69859200
C	-4.23576000	-2.86095800	-0.73037300
C	-3.32731100	-2.14438000	-1.48848100
H	-3.45364300	-1.86550200	2.49067500
H	-5.02197000	-3.35231200	1.23162000
H	-4.93902700	-3.52401500	-1.22448100
H	-3.28548400	-2.21666200	-2.56991000
B	-0.78973300	0.12829000	-0.10719800
C	2.69117600	-1.56727900	0.24196200
C	3.04385400	-0.38568300	-0.40972200
C	3.62791000	-2.52868100	0.58251100
C	4.35227600	-0.09809200	-0.76001800
C	4.96014700	-2.25210300	0.23393000
H	3.34602100	-3.44566300	1.09065400
C	5.31430200	-1.06411500	-0.42236100

H	4.61753600	0.82378600	-1.26833500
H	5.73138200	-2.97658200	0.47924200
H	6.35497600	-0.88445600	-0.67661300
B	0.85244000	-0.37003600	-0.07064600
O	-1.58541800	-0.36724200	1.08964800
O	-1.51486000	-0.52285800	-1.27032000
O	1.32507600	-1.56912600	0.45924400
O	1.90309400	0.37035900	-0.61276500
O	-1.00515700	1.57832600	-0.29160500
C	-0.50802500	2.55062800	0.42224900
N	-0.64229700	3.75301200	-0.08352600
C	-1.29948100	3.93139400	-1.38857200
H	-1.24944200	4.98824600	-1.64832200
H	-0.78648800	3.34099200	-2.15091800
H	-2.34497200	3.61756100	-1.32811400
C	-0.18691400	4.97381200	0.58861000
H	-1.05009300	5.62535400	0.75253600
H	0.28541500	4.74845600	1.54124600
H	0.53093800	5.48112500	-0.06193300
C	0.14861200	2.30981300	1.74466800
H	-0.37154200	2.87046100	2.52832900
H	0.11813800	1.25562500	2.01289000
H	1.19086600	2.64333800	1.71981100

XYZ coordinate of fragmented B2cat2˙1DMA positive charge

B3LYP-D3BJ/6-31+g(d)/SMD-DMA

C	0.42638900	2.57314500	-0.75075700
C	0.60778600	2.52659800	0.63172200
C	0.02325000	3.44133100	1.48710700
C	-0.76931600	4.43258700	0.88432600
C	-0.95192500	4.48091300	-0.50467400
C	-0.35133000	3.54113500	-1.35892700
H	0.16852600	3.39378000	2.56150600
H	-1.25077000	5.17608600	1.51268300
H	-1.57315900	5.26115300	-0.93452800
H	-0.49204200	3.56863700	-2.43463400

B	1.68759800	0.85135900	-0.27267300
C	-2.57502400	-2.10496000	-0.10363400
C	-3.03314400	-0.78972200	-0.03359400
C	-3.43451700	-3.18844500	-0.09861600
C	-4.37790000	-0.47740800	0.04615300
C	-4.80485500	-2.88930900	-0.01815700
H	-3.07064500	-4.20942900	-0.15366500
C	-5.26521400	-1.56653600	0.05239700
H	-4.72530000	0.54931700	0.10065300
H	-5.52196100	-3.70501900	-0.01066500
H	-6.33264000	-1.37555400	0.11355100
B	-0.83942900	-0.75872100	-0.14397900
O	1.42584000	1.44414200	0.94633600
O	1.12105600	1.52320700	-1.33779900
O	-1.17719900	-2.09306900	-0.17417400
O	-1.93235700	0.07356000	-0.05909800
O	2.53083500	-0.21557700	-0.51362600
C	3.00911200	-1.13407800	0.32055400
N	3.95195400	-1.89294300	-0.15502400
C	4.45275800	-1.78686000	-1.53477500
H	5.46974200	-1.38579500	-1.50396800
H	4.46976300	-2.79358400	-1.95828800
H	3.81235400	-1.14208000	-2.13026400
C	4.58733000	-2.93642700	0.67170200
H	5.53975700	-3.18774500	0.20635300
H	4.76737500	-2.56753600	1.68098000
H	3.94700600	-3.82281200	0.69718800
C	2.47412700	-1.27533100	1.70189200
H	2.49578700	-2.31752400	2.02269100
H	3.09146000	-0.68475800	2.39164600
H	1.44966900	-0.90762600	1.75774500

Lewis's structure of Bcat with 1 DMA solvent molecules

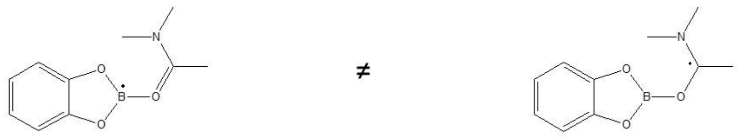

Trigonal pyramidal on Boron

19.2 kcal/mol

Trigonal planar on Boron

0 kcal/mol

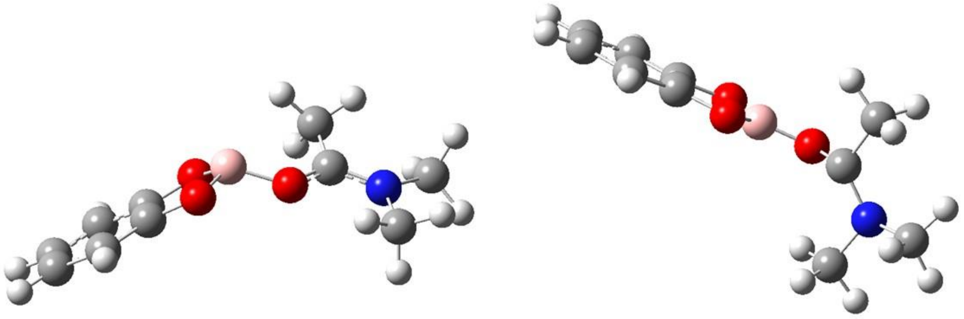

Fukui function for radical $f^0(r)$

Iso=0.01

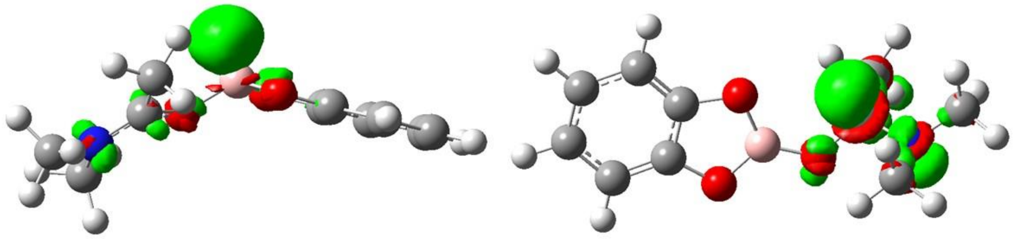

The structure of Bcat with 1 DMA molecules had two Lewis structures. One would be spin density located on boron and one would be spin density located on carbon. So, the Fukui function for radical affinity demonstrates a difference. When the spin density is located on boron, it also prefers to bond with radical. When the spin density is located on carbon, it also prefers to bond with radicals. The geometry of the structure is also demonstrated difference with respect to the boron center. Although the trigonal planar structure is lower energy form, the trigonal pyramidal is still dominant as the structure of this resembles the geometry of B_2cat_2 with 2 DMA complex.

The Fukui function of B_2pin_2

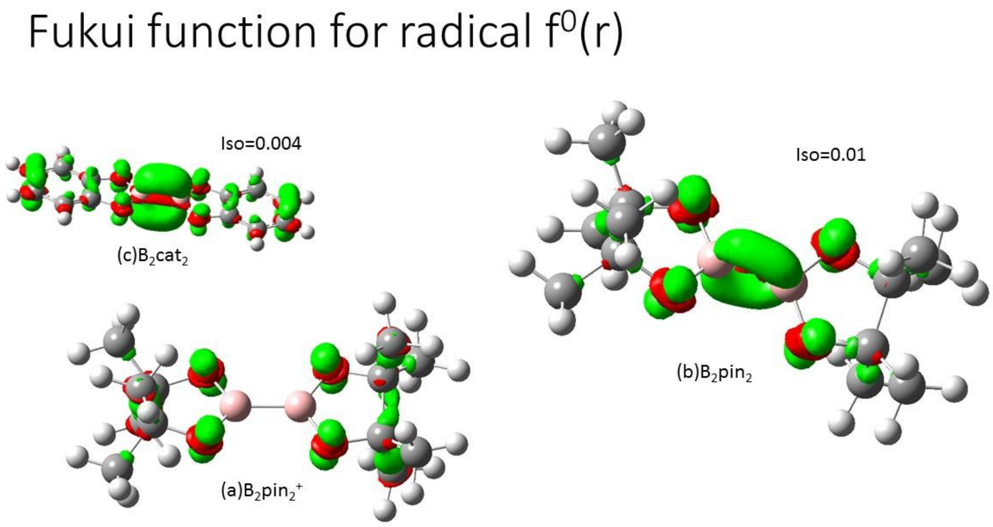

There are 2 target molecules to react with the carbon radical, B_2pin_2 cation radical and B_2pin_2 neutral molecules. The cation radical is generated by quenching triplet state aryl NHPI-ester. We originally thought that cation radical would be the structure to bond with carbon radical. But the plot of Fukui function of B_2pin_2 cation radical disagree that would react with the carbon radical. The radical affinity was located on the oxygen atom.

Then, we move to consider B_2pin_2 neutral molecules as the reaction conditions of Glorius and co-worker had did their reaction in excess. So that we plot the Fukui function of B_2pin_2 neutral molecules. The result shows that the region is bridge between two boron. It cannot select bond with either one of the boron when the radical attack. Therefore, we proposed the structure of BC-8 in the manuscript. The lone pair electrons of pyridine attack the empty p orbital of boron to break carbon-boron bond.

Then, it also raised questions about the free B_2cat_2 and aliphatic carbon radical mechanisms. In fact, the plot of Fukui function of free B_2cat_2 looks identical with B_2pin_2. We also interpret the NMR and existence of free B_2cat_2 in the solution. We try to calculate structures that are similar to BC-8.

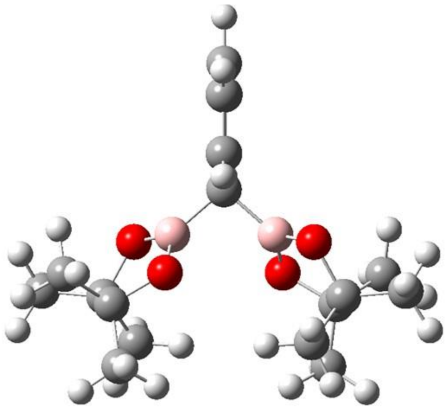

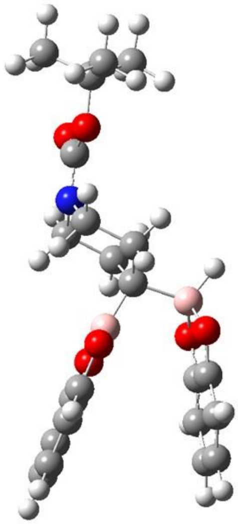

The right panel shows the optimized structure. The hydrogen attached to the Boc aliphatic carbon radical is automatic migrate to the boron compound. However, the reaction can proceed with tertiary carbon. So that the carbon radical would prefer to bond with the Bcat DMA radical rather than free B_2cat_2.

XYZ coordinate of BA-1

C	-5.36483300	-2.05652300	-0.31346200
C	-5.85706800	-0.62393200	-0.51303800
H	-4.49686200	-2.25324500	-0.95130300
H	-5.08904200	-2.24678800	0.72571600
H	-6.16241900	-2.75301900	-0.59686700
C	-7.03044100	-0.27034700	0.39981900
C	-6.22461700	-0.38403100	-1.97621100
H	-7.30237100	0.78526400	0.28244000
H	-7.89838700	-0.87808400	0.11903200
H	-6.79563400	-0.46104900	1.44875500
H	-7.05172200	-1.04271500	-2.26174500
H	-6.53812300	0.65406000	-2.13385400
H	-5.37155200	-0.59529200	-2.63066100
O	-4.74255100	0.32459500	-0.30569600
C	-4.11177600	0.43459800	0.88197400
O	-4.39962600	-0.20034600	1.89515100
C	-2.56592100	1.88149400	-0.42858800
C	-2.15861200	1.42647000	1.93890500
C	-1.36136400	1.02878000	-0.85385200
H	-2.25560900	2.92053100	-0.26529700
H	-3.33525600	1.87120100	-1.19731300
C	-0.93138100	0.56085500	1.61431100
H	-1.86102200	2.47182400	2.08194200
H	-2.64427700	1.07503100	2.84803800
C	-0.34073600	1.05076800	0.28698800
H	-0.91881300	1.43456800	-1.76904700
H	-1.70836300	0.01574700	-1.05828400
H	-0.18482900	0.65440200	2.41125900
H	-1.24724200	-0.48119900	1.55672500
H	-0.07906600	2.09440200	0.44655000
N	-3.12062600	1.38316900	0.83537100
N	0.98942400	0.40413700	-0.02845800
C	1.15857500	-0.95773500	-0.07592400
C	2.09283000	1.22310200	-0.09682000
C	2.43422400	-1.50022700	-0.09158400
C	3.37023200	0.68693000	-0.11012500

C	3.58214000	-0.69641100	-0.07797900
H	2.51316300	-2.57895600	-0.14654300
H	4.20145700	1.38075500	-0.13288900
C	1.94443900	2.70087600	-0.15639500
C	2.44292700	3.48652400	0.89205000
C	1.38120100	3.31810600	-1.28398600
C	2.35428800	4.87860000	0.82451900
H	2.88515100	3.00612100	1.76026600
C	1.30247300	4.70929800	-1.34981400
H	1.01238500	2.70992700	-2.10434000
C	1.78313400	5.49160400	-0.29438700
H	2.73240100	5.48184100	1.64525000
H	0.86798800	5.18159600	-2.22652600
H	1.71677900	6.57490300	-0.34685100
C	0.01486300	-1.91040700	-0.14897400
C	-0.29348700	-2.71439800	0.95700600
C	-0.65670700	-2.11259500	-1.36210600
C	-1.30253500	-3.67441700	0.86502600
H	0.24409500	-2.57265100	1.89016600
C	-1.65883700	-3.08051000	-1.45335200
H	-0.39028800	-1.51969400	-2.23181500
C	-1.99053300	-3.85515700	-0.33849400
H	-1.54930000	-4.27974500	1.73286100
H	-2.17893000	-3.22695900	-2.39584500
H	-2.77811200	-4.60030100	-0.40879300
C	4.93561700	-1.28094100	-0.05464100
C	5.16230200	-2.51616300	0.58019900
C	6.01941700	-0.61552900	-0.65651500
C	6.44216600	-3.06838800	0.61548200
H	4.34303800	-3.03380700	1.07014100
C	7.29584500	-1.17650100	-0.62876100
H	5.86347100	0.32754600	-1.17143600
C	7.51206000	-2.40228300	0.00905600
H	6.60466200	-4.01668200	1.12032800
H	8.12088600	-0.65811600	-1.10938800
H	8.50825000	-2.83561500	0.03332900

XYZ coordinate of BA-2

C	5.07818400	-1.99927100	-0.04532400
C	5.68151800	-0.65280900	-0.44319100
H	4.37172300	-1.87093300	0.78164100
H	4.55933700	-2.47164500	-0.88202300
H	5.87934300	-2.66768900	0.29083400
C	6.60959300	-0.75640400	-1.65283700
C	6.41320000	-0.02533200	0.74217600
H	6.96379800	0.23776600	-1.95001700
H	7.48301700	-1.36219500	-1.38469100
H	6.10875600	-1.22247200	-2.50356200
H	7.25151000	-0.66423800	1.03982800
H	6.80728900	0.96230800	0.47732700
H	5.73981800	0.08518400	1.59947600
O	4.60427400	0.32263600	-0.70373200
C	3.68108600	0.12613000	-1.67000000
O	3.69049200	-0.80724300	-2.47166100
C	2.54047500	2.01195200	-0.52062500
C	1.51616400	0.91372800	-2.44696800
C	1.52007900	1.39155900	0.44320600
H	2.16989700	2.97008800	-0.90516100
H	3.49167400	2.19035000	-0.02359300
C	0.42927700	0.29039500	-1.56076800
H	1.18547200	1.88998300	-2.82120800
H	1.74072500	0.27459400	-3.30034900
C	0.20663000	1.15212500	-0.30786800
H	1.35343700	2.06060700	1.29439200
H	1.93557400	0.45693900	0.82443400
H	-0.50719400	0.20847900	-2.12559700
H	0.73966400	-0.72081600	-1.27452700
H	-0.13796300	2.13131700	-0.64789400
N	2.74494900	1.12849800	-1.67650400
N	-0.93348300	0.61971500	0.51692000
C	-0.96961900	-0.76922900	0.77802900
C	-2.17807900	1.25024700	0.27722300
C	-2.08473100	-1.50839900	0.47920800
C	-3.30078800	0.50646100	0.00043600

C	-3.28649100	-0.91423400	-0.00931800
H	-2.06469800	-2.56328100	0.73201200
H	-4.22542500	1.04540000	-0.16981400
C	-2.22251600	2.71877600	0.39250100
C	-3.07383400	3.49088400	-0.42263200
C	-1.41870800	3.38671500	1.33859300
C	-3.12976100	4.87785500	-0.28450700
H	-3.67793300	3.00305800	-1.18228900
C	-1.47457100	4.77349100	1.47257100
H	-0.75914100	2.80759900	1.97704700
C	-2.33075200	5.52854700	0.66277600
H	-3.78992500	5.45390600	-0.92814600
H	-0.85112000	5.26580000	2.21490800
H	-2.37112500	6.60975600	0.76488300
C	0.18370500	-1.38459300	1.46761600
C	0.73702400	-2.59318900	1.01072500
C	0.71587400	-0.79523400	2.62897000
C	1.79409800	-3.19569200	1.69592800
H	0.34794100	-3.04854300	0.10415400
C	1.76797100	-1.40003800	3.31531500
H	0.29604800	0.13915100	2.98988700
C	2.31481000	-2.60204600	2.84994900
H	2.22064000	-4.12178200	1.31924900
H	2.16322300	-0.93317400	4.21404800
H	3.14216700	-3.06770000	3.37882800
C	-4.45172600	-1.71601700	-0.38018900
C	-4.32451600	-3.09834000	-0.66276100
C	-5.74764300	-1.15418700	-0.48680600
C	-5.42737300	-3.87162300	-1.02118300
H	-3.34730900	-3.56932300	-0.62320200
C	-6.84730100	-1.93049800	-0.84749500
H	-5.90481300	-0.10279400	-0.26850300
C	-6.70049500	-3.29702000	-1.11698500
H	-5.28950000	-4.92874200	-1.23620900
H	-7.82834700	-1.46570200	-0.91238200
H	-7.55966500	-3.90014200	-1.39813100

XYZ coordinate of BA-3

C	2.48172100	-0.16403800	1.60879200
C	2.41340800	0.19848900	0.12532300
H	1.90790600	0.55521900	2.20495300
H	2.09356600	-1.16734300	1.79483400
H	3.52566900	-0.12500900	1.94077300
C	3.12285700	-0.82525300	-0.76067700
C	2.98369100	1.59639100	-0.10976100
H	2.99624800	-0.56831800	-1.81896100
H	4.19538500	-0.81514500	-0.53429600
H	2.74038000	-1.83403800	-0.59308900
H	4.04161400	1.61684800	0.17305300
H	2.90180200	1.87729100	-1.16580200
H	2.44953500	2.34027600	0.49198300
O	1.00794600	0.35300100	-0.29693900
C	0.12938900	-0.67413800	-0.24645400
O	0.40001900	-1.80987400	0.14577600
C	-1.49026700	1.08748300	-0.96266500
C	-2.23440500	-1.20849300	-0.53188400
C	-2.28756900	1.63660200	0.23639800
H	-2.12041000	1.09708000	-1.86043700
H	-0.60394700	1.68762800	-1.15803700
C	-3.07275600	-0.77151700	0.68579200
H	-2.84719900	-1.16591800	-1.44027800
H	-1.85971000	-2.22432100	-0.41045000
C	-3.37526400	0.69276100	0.64118200
H	-2.68922300	2.62746900	-0.00966800
H	-1.57556400	1.78345100	1.07119700
H	-3.99212000	-1.36816400	0.73539400
H	-2.49050600	-1.01452400	1.59535700
H	-4.27342600	1.07512200	1.12028500
N	-1.09836500	-0.30462000	-0.72054200

XYZ coordinate of BA-4

N	-1.32356000	0.01818200	-0.05143600
C	-0.62604800	1.16786900	-0.01261600
C	-0.65902100	-1.15168400	-0.05929700
C	0.77440900	1.18539500	0.02920000
C	0.74053600	-1.20995600	-0.02378600
C	1.48261800	-0.02296500	0.02062300
H	1.30607700	2.12608100	0.11118800
H	1.24812400	-2.16728200	-0.02645100
C	-1.48517900	-2.38834700	-0.10098800
C	-0.98819300	-3.58422000	-0.64749700
C	-2.79373500	-2.37687800	0.41254700
C	-1.77620500	-4.73681900	-0.67426800
H	0.00980000	-3.61559300	-1.07408500
C	-3.57888800	-3.53021900	0.39029000
H	-3.18671700	-1.45910500	0.83759500
C	-3.07361400	-4.71611700	-0.15333500
H	-1.37711100	-5.64939400	-1.10943100
H	-4.58537100	-3.50375200	0.80015100
H	-3.68576000	-5.61401000	-0.17304700
C	-1.41477100	2.42876600	0.00070800
C	-0.88397800	3.62723900	-0.50681400
C	-2.71941000	2.43902100	0.52366800
C	-1.63587200	4.80381800	-0.48716900
H	0.11171300	3.64156800	-0.93979400
C	-3.46857500	3.61605700	0.54764600
H	-3.13768300	1.51924500	0.91934000
C	-2.93011200	4.80426200	0.04215700
H	-1.21168200	5.71861800	-0.89292700
H	-4.47274000	3.60634200	0.96387100
H	-3.51451800	5.72052000	0.05804100
C	2.96495500	-0.04386900	0.07328900
C	3.71839300	0.95050800	-0.57515800
C	3.64602600	-1.05704800	0.77054500
C	5.11347400	0.93089500	-0.52862400
H	3.21234600	1.73111700	-1.13607300
C	5.04102700	-1.07334000	0.82038600

H	3.08311000	-1.82363300	1.29525300
C	5.78048200	-0.08027900	0.17015400
H	5.67906800	1.70301200	-1.04372200
H	5.54977600	-1.85927200	1.37251100
H	6.86658500	-0.09427600	0.20767700

XYZ coordinate of product of BA-2

C	-6.00311300	0.48515000	1.04410900
C	-5.88638000	-0.21251400	-0.31057700
H	-5.36101800	-0.00335500	1.78329000
H	-5.72899400	1.53928900	0.97550500
H	-7.04008000	0.41284700	1.39302200
C	-6.75044000	0.45247700	-1.38216900
C	-6.24074000	-1.69281600	-0.17750800
H	-6.59465700	-0.03038800	-2.35413000
H	-7.80719800	0.34260400	-1.11286300
H	-6.52557800	1.51729400	-1.47554400
H	-7.27615300	-1.79675000	0.16439900
H	-6.14007300	-2.20624500	-1.14033800
H	-5.58457500	-2.18305200	0.55040300
O	-4.48157400	-0.26194100	-0.75903300
C	-3.79409400	0.85862000	-1.07997400
O	-4.17841100	2.00553500	-0.84295400
C	-2.21050500	-0.77374300	-2.13278100
C	-1.76104300	1.64410600	-2.19088400
C	-0.77618400	-1.08559900	-1.67858000
H	-2.26635600	-0.79860500	-3.23159600
H	-2.90837700	-1.50771400	-1.73703900
C	-0.30446900	1.44182000	-1.74847100
H	-1.81990200	1.64540200	-3.28986700
H	-2.15357600	2.59176800	-1.82459000
C	0.16300400	0.03984700	-1.97036800
H	-0.43950300	-2.01555600	-2.15420900
H	-0.80195800	-1.28535300	-0.59322800
H	0.33806300	2.16198400	-2.27069000
H	-0.23811700	1.69249600	-0.67546700

H	1.22946500	-0.16002600	-2.03585800
N	-2.60749100	0.56391900	-1.68400600
N	1.66133000	-1.34255800	0.89856900
C	1.04555800	-0.23400600	1.34702300
C	2.85034100	-1.22687800	0.28224800
C	1.61842800	1.03693500	1.21559400
C	3.47190500	0.01479200	0.09465700
C	2.85236700	1.17732300	0.56960300
H	1.12207500	1.90514600	1.63205000
H	4.42604400	0.07554000	-0.41546500
C	3.47189900	-2.48623100	-0.20780800
C	4.34942700	-2.49008000	-1.30558900
C	3.17914000	-3.70743900	0.42346100
C	4.92050800	-3.68191800	-1.75678300
H	4.57307300	-1.56429200	-1.82720200
C	3.75447100	-4.89736300	-0.02395500
H	2.50239600	-3.71480100	1.27179000
C	4.62800000	-4.89021000	-1.11677400
H	5.58888400	-3.66604600	-2.61384700
H	3.52257500	-5.83085800	0.48261700
H	5.07375300	-5.81724200	-1.46780400
C	-0.29164900	-0.42089600	1.96769100
C	-1.24856600	0.60871700	1.96025700
C	-0.63818900	-1.65622200	2.54104700
C	-2.51301600	0.40978600	2.51584900
H	-1.02177300	1.56258600	1.49548100
C	-1.90104200	-1.85326200	3.10072200
H	0.09141200	-2.45938400	2.54673500
C	-2.84477400	-0.82083400	3.08974700
H	-3.24390700	1.21257700	2.48440500
H	-2.14882500	-2.81364400	3.54578800
H	-3.83142500	-0.97629000	3.51789100
C	3.47395400	2.51174700	0.38976800
C	2.67367300	3.64515900	0.16157500
C	4.86991600	2.67127500	0.43479500
C	3.25350700	4.90210300	-0.01843800
H	1.59433600	3.53952800	0.10420400
C	5.44887200	3.92935400	0.25923200

H	5.50479200	1.81143500	0.62848900
C	4.64334400	5.04951200	0.03036600
H	2.61915900	5.76509600	-0.20350900
H	6.52958000	4.03509100	0.30703800
H	5.09477600	6.02837200	-0.10841800

XYZ coordinate of transition state BA-2

C	5.13185300	-1.85291900	0.25447700
C	5.64575100	-0.65472300	-0.54180400
H	4.53510700	-1.51455400	1.10758100
H	4.52094300	-2.51421600	-0.36332600
H	5.98605300	-2.42242300	0.63891900
C	6.43125200	-1.06689800	-1.78627700
C	6.49032800	0.25497200	0.34873200
H	6.71880800	-0.18322600	-2.36810000
H	7.34740900	-1.58341200	-1.47735000
H	5.85113000	-1.73721100	-2.42351800
H	7.37185900	-0.28984700	0.70322800
H	6.82812700	1.13790100	-0.20550900
H	5.91487500	0.58765100	1.21981000
O	4.51923500	0.22431700	-0.91547700
C	3.52258000	-0.19552900	-1.72394400
O	3.44353000	-1.31996000	-2.21760500
C	2.54336400	2.01758900	-1.13971300
C	1.34717900	0.45600200	-2.59853500
C	1.51148300	1.81257700	-0.00062200
H	2.22807300	2.84236300	-1.78917100
H	3.52767900	2.25124700	-0.73801900
C	0.25902200	0.22318800	-1.51965400
H	1.05628300	1.28569200	-3.25246700
H	1.48858400	-0.43748600	-3.20654400
C	0.20213900	1.41066900	-0.60884500
H	1.41803000	2.73408100	0.58473100
H	1.89540500	1.02629400	0.65735400
H	-0.70661200	0.04330200	-2.00569500
H	0.53289900	-0.68036200	-0.96000200

H	-0.40973300	2.23700800	-0.96198200
N	2.61898200	0.80778500	-1.96514600
N	-1.17861900	0.85506600	0.89634300
C	-0.97543000	-0.46927700	1.19385300
C	-2.40449800	1.20251200	0.37640100
C	-1.87451800	-1.45967800	0.81773200
C	-3.33997900	0.24679300	-0.00502600
C	-3.08285700	-1.12611000	0.17414500
H	-1.67390000	-2.48864500	1.09606300
H	-4.28626400	0.56825600	-0.42389900
C	-2.65625400	2.65399800	0.22744800
C	-3.59441800	3.15725100	-0.69424300
C	-1.93299300	3.57530300	1.00920000
C	-3.80891100	4.53031100	-0.81931900
H	-4.14525400	2.47745700	-1.33712900
C	-2.14789700	4.94797200	0.88330000
H	-1.20301300	3.20153200	1.71917500
C	-3.08822100	5.43446400	-0.03103200
H	-4.53348600	4.89540000	-1.54289800
H	-1.58062800	5.63928900	1.50178800
H	-3.25456900	6.50380000	-0.13200000
C	0.25646100	-0.79320700	1.94857400
C	0.94364600	-2.00306500	1.74400600
C	0.76424200	0.11489700	2.89457500
C	2.10088000	-2.29661500	2.46655300
H	0.58670300	-2.70596500	0.99669900
C	1.91876900	-0.18024400	3.61964800
H	0.24485800	1.05449000	3.05448400
C	2.59433900	-1.38728200	3.40796400
H	2.62754800	-3.22909400	2.28130100
H	2.29350900	0.53308700	4.34959700
H	3.49932300	-1.61416100	3.96548400
C	-4.04462300	-2.16434900	-0.24490000
C	-3.59642200	-3.44971000	-0.61156000
C	-5.42989100	-1.90840300	-0.29733500
C	-4.49603400	-4.43862900	-1.01171900
H	-2.53279800	-3.66994900	-0.60521100
C	-6.32824100	-2.89779800	-0.69838900

H	-5.81297700	-0.93665000	-0.00020200
C	-5.86789700	-4.16929700	-1.05829600
H	-4.12276400	-5.41917500	-1.29673600
H	-7.39253700	-2.67694100	-0.72144300
H	-6.56855400	-4.93894500	-1.37111500

XYZ coordinate of BA-5

C	4.43685900	2.35971100	1.38576900
C	5.22754800	1.64737100	0.28869000
H	3.45526300	2.67062200	1.00919400
H	4.29463900	1.71948300	2.25858200
H	4.98259300	3.25836900	1.69573700
C	6.56917800	1.11097000	0.78673800
C	5.43187300	2.57251800	-0.90981300
H	7.07654900	0.55175200	-0.00816100
H	7.20954600	1.95391200	1.07137800
H	6.44395900	0.45955200	1.65391000
H	6.02605200	3.44190000	-0.60867900
H	5.96164900	2.05321200	-1.71630300
H	4.46951800	2.92740400	-1.29547400
O	4.42227300	0.55229900	-0.28825600
C	4.00856700	-0.49766400	0.45479500
O	4.25747900	-0.65446000	1.65016200
C	2.78892800	-1.09877500	-1.64014700
C	2.57609600	-2.47044700	0.37989400
C	1.31212500	-0.69478300	-1.57783800
H	2.90188200	-2.00745300	-2.24505300
H	3.38696600	-0.31118200	-2.09323400
C	1.09026400	-2.12052600	0.51595900
H	2.69422000	-3.38419500	-0.21582700
H	3.02589000	-2.63378300	1.35845600
C	0.49791100	-1.80551600	-0.87712300
H	0.93154900	-0.53187000	-2.59285400
H	1.21790500	0.24684700	-1.02562100
H	0.55323000	-2.95991900	0.97287100
H	0.98286400	-1.25130100	1.17447900

H	0.54574900	-2.71288400	-1.48943300
N	3.29969400	-1.39389600	-0.29804900
C	-0.96422700	-1.46437400	-0.79231900
O	-1.89862500	-2.06402200	-1.25823700
O	-1.13941300	-0.28554400	-0.05145900
C	-3.11866900	0.86425600	-0.88577700
C	-3.28174600	-0.41056400	1.10047600
C	-4.51053200	0.95803700	-0.36691600
C	-4.60717400	0.20146600	0.81178800
C	-5.60290900	1.64164400	-0.87904500
C	-5.79870000	0.10523700	1.51445900
C	-6.81340400	1.54773100	-0.17404900
H	-5.52803400	2.22645800	-1.79055300
C	-6.90963600	0.79335300	1.00134200
H	-5.87222300	-0.48028400	2.42559700
H	-7.68993300	2.06876200	-0.54775300
H	-7.85948900	0.73865100	1.52490700
N	-2.45401000	0.08531800	0.07780300
O	-2.60720100	1.32356400	-1.88701100
O	-2.92504300	-1.16207300	1.98533400

XYZ coordinate of BA-6

C	4.36953300	2.37500900	1.38388900
C	5.18240800	1.67223100	0.29676700
H	3.38593600	2.66686200	0.99752200
H	4.23083400	1.73586800	2.25807300
H	4.89655100	3.28443300	1.69494500
C	6.52902000	1.16215100	0.80916500
C	5.38129900	2.59662600	-0.90334600
H	7.05341200	0.60860400	0.02133100
H	7.15179700	2.01755300	1.09577600
H	6.40745100	0.51224100	1.67798200
H	5.95784500	3.47722000	-0.60044600
H	5.92679900	2.08360500	-1.70338800
H	4.41635300	2.93345800	-1.29855900
O	4.40219600	0.56147000	-0.28311100
C	3.99588400	-0.49121000	0.46120600

O	4.24136900	-0.64088900	1.65843100
C	2.79282200	-1.11024100	-1.63757100
C	2.58261500	-2.47777200	0.38598500
C	1.31229300	-0.71975100	-1.58152600
H	2.91632700	-2.02043400	-2.23838000
H	3.38727500	-0.31989400	-2.09080200
C	1.09352700	-2.13825900	0.51307700
H	2.71133400	-3.39183500	-0.20734500
H	3.03083800	-2.63524400	1.36633000
C	0.50143400	-1.83153300	-0.88002600
H	0.93497700	-0.56243800	-2.59894900
H	1.20703200	0.22341500	-1.03371300
H	0.56151600	-2.97993100	0.97231000
H	0.97674300	-1.26830500	1.16897800
H	0.56018200	-2.74015700	-1.49003500
N	3.30141000	-1.39726900	-0.29227700
C	-0.96756900	-1.49270400	-0.80799000
O	-1.87594100	-2.09780700	-1.32645200
O	-1.15620700	-0.35575400	-0.04558100
C	-3.09167300	0.88902800	-0.86902500
C	-3.31512400	-0.46907200	1.09494200
C	-4.44285700	0.98842500	-0.37009200
C	-4.57722300	0.17182400	0.81063200
C	-5.54523400	1.71748000	-0.85224800
C	-5.81118000	0.10149900	1.48267700
C	-6.75615100	1.63481600	-0.17609600
H	-5.44845500	2.33491200	-1.74249100
C	-6.88905200	0.82730700	0.99102400
H	-5.91687100	-0.51352200	2.37358600
H	-7.61658700	2.19185600	-0.53870300
H	-7.84869000	0.78118000	1.50002500
N	-2.47458800	0.03231200	0.07293200
O	-2.50865800	1.38721100	-1.85514700
O	-2.94513200	-1.26671500	1.98219900

XYZ coordinate of product of BA-6

C	5.44653200	1.21754000	1.83539700
C	5.87022900	0.95844500	0.38964200
H	4.52476400	1.81045900	1.86306100
H	5.28347000	0.28491800	2.37906600
H	6.23298300	1.78688600	2.34429900
C	7.10203200	0.05845500	0.29283000
C	6.11400400	2.27863200	-0.34010100
H	7.33556400	-0.15860400	-0.75619600
H	7.96200500	0.57703200	0.73246500
H	6.95320600	-0.88338800	0.82458700
H	6.93621900	2.82043700	0.13954600
H	6.38109800	2.10021600	-1.38782500
H	5.21895200	2.91008200	-0.31067200
O	4.74984900	0.37588300	-0.37276900
C	4.21034800	-0.82173800	-0.04857300
O	4.58951400	-1.53026200	0.88517000
C	2.59653900	-0.23629100	-1.86430000
C	2.35967700	-2.31802500	-0.58685400
C	1.26542200	0.29356000	-1.31858600
H	2.42455500	-0.78775300	-2.79814600
H	3.29006000	0.57577000	-2.07286100
C	1.01913900	-1.85123500	-0.00668400
H	2.19264200	-2.88260700	-1.51340500
H	2.89275200	-2.95783400	0.11586900
C	0.32634400	-0.87463600	-0.97400100
H	0.80028600	0.94843900	-2.06647500
H	1.45722100	0.89442800	-0.42174800
H	0.37894600	-2.72304700	0.17891600
H	1.19709400	-1.35625700	0.95567500
H	0.08921200	-1.41456000	-1.89934300
N	3.20795200	-1.16810600	-0.90962000
C	-1.03881700	-0.39660000	-0.42272700
O	-2.08677800	-0.72989300	-0.99466100
O	-0.92186300	0.33170300	0.63523800
C	-3.55104000	1.62578300	0.27975900
C	-3.67778300	-0.24537700	1.55296200
C	-4.83036300	0.93018800	-0.09478600
C	-4.90887200	-0.22157200	0.69013900

C	-5.82525300	1.25623700	-1.00690100
C	-5.98577300	-1.09287700	0.59524300
C	-6.91518400	0.37850300	-1.11843600
H	-5.76492100	2.15406100	-1.61579000
C	-6.99441700	-0.77836400	-0.32913000
H	-6.04747200	-1.98754200	1.20858900
H	-7.71074500	0.59668200	-1.82593500
H	-7.85011500	-1.43968400	-0.43643600
N	-2.96560900	0.93028000	1.33378300
O	-3.12261200	2.66092100	-0.22549000
O	-3.37945700	-1.12924100	2.35304500

XYZ coordinate of transition state of BA-6

C	-3.56620400	2.91001300	-0.63597400
C	-4.62258600	2.07167800	0.08387600
H	-2.61432500	2.87373600	-0.09327800
H	-3.40434400	2.56099700	-1.65771000
H	-3.89828100	3.95404900	-0.67115300
C	-5.94495300	2.01331900	-0.68059900
C	-4.84640100	2.59664000	1.50124000
H	-6.65389400	1.35180000	-0.16886000
H	-6.38311100	3.01748500	-0.71820700
H	-5.80206100	1.65653200	-1.70249300
H	-5.23028600	3.62160900	1.45986800
H	-5.57393000	1.97544200	2.03574800
H	-3.90792800	2.60055200	2.06691300
O	-4.11100600	0.70901200	0.32659800
C	-3.76547800	-0.11607500	-0.68759200
O	-3.86357100	0.16324900	-1.88293500
C	-2.95998500	-1.56399300	1.17945400
C	-2.67835600	-2.24781600	-1.15732200
C	-1.44422600	-1.44234700	1.37083200
H	-3.28778600	-2.58023100	1.43387900
H	-3.49388400	-0.86446500	1.81931500
C	-1.15296100	-2.14696600	-1.04585500
H	-3.01023300	-3.26340000	-0.90634700

H	-3.01472600	-2.01422700	-2.16694300
C	-0.70634700	-2.39597500	0.40688000
H	-1.18711400	-1.68021700	2.41036300
H	-1.13743600	-0.40748100	1.17757500
H	-0.68640100	-2.87954800	-1.71607400
H	-0.83485700	-1.14931500	-1.36875700
H	-0.96704000	-3.42571400	0.68076100
N	-3.32326400	-1.32312600	-0.22137000
C	0.80637800	-2.30043100	0.59973000
O	1.46365900	-3.17021600	1.14913300
O	1.27167900	-1.15097200	0.11163100
C	3.07608100	0.10947600	1.19344800
C	3.51650000	-0.68504200	-0.96303000
C	4.10855800	0.97083000	0.59505800
C	4.32841600	0.49327400	-0.72734900
C	4.77813600	2.07510400	1.10527500
C	5.31170300	1.11203600	-1.52742500
C	5.73212900	2.70707500	0.29175300
H	4.58167500	2.42853400	2.11445400
C	5.98970900	2.21416600	-1.00687400
H	5.52260300	0.75155500	-2.53039100
H	6.28470300	3.56514100	0.66348200
H	6.73648900	2.71406000	-1.61995800
N	2.88152000	-0.97094300	0.27194200
O	2.46297200	0.24028500	2.24445100
O	3.43797900	-1.41613500	-1.98254700

XYZ coordinate of BA-7

C	3.04927500	-1.01716100	-1.56154400
C	3.20927200	-0.55795300	-0.11256600
H	2.27753400	-1.79246900	-1.63426000
H	2.78083100	-0.18756900	-2.21856500
H	3.99634600	-1.44624700	-1.90862000
C	4.20610600	0.59143100	0.03172200
C	3.61023200	-1.73127500	0.77977100
H	4.24512700	0.93608600	1.07173200

H	5.20536000	0.23726200	-0.24696200
H	3.94333200	1.43384100	-0.61125000
H	4.58445000	-2.11966700	0.46413900
H	3.68552100	-1.41591300	1.82649000
H	2.87535000	-2.54114100	0.71033800
O	1.89815700	-0.16859700	0.44527600
C	1.17709800	0.85095500	-0.06887300
O	1.52167600	1.54545800	-1.02474200
C	-0.51358700	0.08652200	1.60432300
C	-0.99723400	1.94780500	0.08762000
C	-1.61211100	-0.77784600	0.97418100
H	-0.92993300	0.66121200	2.44162200
H	0.29502800	-0.53394800	1.98408500
C	-2.11651700	1.15847200	-0.60082900
H	-1.40970700	2.53256800	0.91935000
H	-0.51986200	2.63034400	-0.61442100
C	-2.71723800	0.12723300	0.38744000
H	-2.03497700	-1.44537900	1.73360400
H	-1.17429300	-1.39704300	0.18272000
H	-2.89440800	1.84823300	-0.94705200
H	-1.70783300	0.63848700	-1.47538400
H	-3.20983400	0.67147400	1.20401100
N	0.01570900	1.04324500	0.63002000
C	-3.77206200	-0.68058500	-0.29273600
O	-5.01880000	-0.53755900	-0.10449400
O	-3.55820800	-1.58910600	-1.14888600

XYZ coordinate of product of BA-7

C	-2.51771800	1.85444200	-0.91179700
C	-2.98064300	0.79836400	0.09163100
H	-1.65017300	2.39894600	-0.52081000
H	-2.25153100	1.40737500	-1.87163300
H	-3.32730500	2.57548400	-1.07381700
C	-4.13035700	-0.05476800	-0.44355200
C	-3.37127900	1.45364300	1.41535800
H	-4.38933800	-0.84190200	0.27434200

H	-5.01274300	0.58001500	-0.58519200
H	-3.87605500	-0.51711900	-1.39935400
H	-4.21801100	2.13014800	1.25746000
H	-3.66478600	0.69733500	2.15199900
H	-2.53578300	2.03341100	1.82351400
O	-1.84624200	-0.06070100	0.48464100
C	-1.18705900	-0.82743600	-0.41179300
O	-1.46094500	-0.89995900	-1.60970400
C	0.30639400	-1.26507100	1.53569200
C	0.77990200	-2.24130700	-0.66463600
C	1.55943700	-0.35479200	1.45851000
H	0.57397300	-2.22176000	1.99910400
H	-0.48189000	-0.80197200	2.12613700
C	2.05428900	-1.37292300	-0.83040900
H	1.03682300	-3.19050500	-0.18064800
H	0.31798900	-2.45061700	-1.62925700
C	2.55751800	-0.93437100	0.50755000
H	1.98135500	-0.22491400	2.46154100
H	1.21618900	0.63408500	1.11236500
H	2.81334900	-1.93500400	-1.38612800
H	1.77021200	-0.50271600	-1.44483100
H	3.51266400	-1.29816900	0.87638000
N	-0.19077500	-1.55237600	0.18700800
C	3.97066700	1.69914600	-0.30318400
O	4.98393300	1.30207000	0.12571900
O	2.98245200	2.14170800	-0.74571300

XYZ coordinate of BA-8

C	1.31163700	1.11523300	0.00017500
C	1.31163800	-1.11523300	0.00030900
C	-0.14351500	0.69747100	0.00002100
C	-0.14351300	-0.69747100	0.00004600
C	-1.32719200	1.42261300	-0.00005000
C	-1.32719000	-1.42261300	-0.00002300
C	-2.53348000	0.70062900	0.00001100
H	-1.32725800	2.50954900	-0.00005100

C	-2.53347900	-0.70063200	0.00002200
H	-1.32725500	-2.50954900	0.00001800
H	-3.48011800	1.23500800	0.00005100
H	-3.48011600	-1.23501400	0.00007400
N	2.11162500	0.00000200	0.00065900
O	1.69649400	2.29387900	-0.00044500
O	1.69649800	-2.29387800	-0.00052600

XYZ coordinate of BA-9

C	-2.87053700	-0.84481400	0.02609900
C	-2.44970600	-1.28668400	-1.24512400
C	-3.35693100	-1.79517000	-2.16450000
C	-4.71563800	-1.85616700	-1.78741700
C	-5.13229700	-1.41613800	-0.52839900
C	-4.20517600	-0.89939800	0.40194900
H	-3.02798100	-2.13155000	-3.14457100
H	-5.44457100	-2.25169100	-2.49045400
H	-6.18375700	-1.46994600	-0.25725600
H	-4.52269800	-0.55070100	1.38148600
B	-0.58885500	-0.63744600	-0.07110600
C	2.87050800	0.84478800	0.02625500
C	2.44976800	1.28658300	-1.24502400
C	4.20512100	0.89938700	0.40219600
C	3.35706100	1.79500600	-2.16436900
C	5.13231000	1.41606500	-0.52811900
H	4.52257300	0.55074800	1.38177500
C	4.71574300	1.85601700	-1.78719400
H	3.02818200	2.13132500	-3.14448400
H	6.18375200	1.46988400	-0.25690600
H	5.44472700	2.25149400	-2.49020400
B	0.58883100	0.63741300	-0.07110300
O	-1.11654900	-1.10124600	-1.39556500
O	-1.82000500	-0.35978500	0.73017000
O	1.81992600	0.35979800	0.73027800
O	1.11662200	1.10114500	-1.39554800
O	0.01881500	-1.90963900	0.65004000

C	1.15226100	-2.44730200	0.38274400
N	1.71461600	-3.19539000	1.32364000
C	1.11230600	-3.30162200	2.65427800
H	0.44475800	-4.17004900	2.70195900
H	1.91547200	-3.42762900	3.38434200
H	0.54767100	-2.39860200	2.88208200
C	2.93501100	-3.97618300	1.11902000
H	2.78478000	-4.97133000	1.54790100
H	3.16200800	-4.08394100	0.06055200
H	3.77696600	-3.49282600	1.62702500
C	1.81266300	-2.29064000	-0.95579200
H	2.86552400	-2.01794800	-0.84870100
H	1.75807800	-3.23765500	-1.50654700
H	1.29893600	-1.53033200	-1.53892500
O	-0.01888700	1.90964100	0.64993200
C	-1.15227500	2.44734600	0.38247900
N	-1.71466400	3.19553100	1.32328000
C	-1.81257400	2.29060200	-0.95610300
C	-1.11243900	3.30183000	2.65394800
C	-2.93501700	3.97636400	1.11854300
H	-2.86543800	2.01789600	-0.84907100
H	-1.75796700	3.23758500	-1.50690800
H	-1.29879800	1.53026600	-1.53915600
H	-0.44501000	4.17034600	2.70166400
H	-1.91565600	3.42772800	3.38397600
H	-0.54769600	2.39888400	2.88177200
H	-2.78479500	4.97150100	1.54745100
H	-3.16190400	4.08414700	0.06005500
H	-3.77704000	3.49303100	1.62645700

XYZ coordinate of BA-10

C	2.09705200	-0.70194600	-0.33436900
C	1.92939600	0.65232900	0.00315100
C	2.96285800	1.39522500	0.55413600
C	4.19329200	0.74025100	0.76540600
C	4.36062400	-0.60655600	0.42917600

C	3.30466900	-1.35362000	-0.13138200
H	2.82545700	2.44114000	0.81452000
H	5.02283600	1.29593000	1.19484500
H	5.31945200	-1.08951000	0.59896100
H	3.42817600	-2.40099800	-0.39285400
B	0.01146200	-0.08636700	-0.93161500
O	0.66236200	1.05996300	-0.29946200
O	0.94439300	-1.20234500	-0.86460400
O	-1.27844300	-0.46526000	-0.33327800
C	-2.33320600	0.27987800	-0.19727300
N	-3.41546100	-0.28867700	0.31244200
C	-3.44558500	-1.71202000	0.66024500
H	-3.27162800	-1.83641400	1.73547500
H	-4.43430400	-2.10334500	0.40881600
H	-2.68551600	-2.25492500	0.10200400
C	-4.62985800	0.47216300	0.62428200
H	-5.11262900	-0.00552300	1.47922900
H	-4.38617900	1.49914900	0.89522800
H	-5.31931900	0.46331400	-0.22695000
C	-2.32057400	1.69387700	-0.66842300
H	-3.31751400	2.05661600	-0.92084000
H	-1.87920800	2.35145200	0.08845900
H	-1.67720600	1.73935200	-1.55686500

XYZ coordinate of BA-11

C	-2.15971800	-0.73643400	0.21973100
C	-1.94756400	0.56019900	-0.24957100
C	-2.98786400	1.40648100	-0.58347900
C	-4.28330800	0.88595700	-0.42382300
C	-4.49755300	-0.41647900	0.04766400
C	-3.42745600	-1.26318600	0.38266800
H	-2.81257000	2.41350700	-0.94817100
H	-5.13590100	1.51063300	-0.67330200
H	-5.51355900	-0.78381900	0.15801300
H	-3.58438700	-2.27280100	0.74865600
B	-0.00774100	-0.37087700	0.13923400

O	-0.57604500	0.80222400	-0.30792900
O	-0.93029500	-1.33797800	0.47021400
O	1.34409800	-0.63980500	0.20337800
C	2.34742200	0.23295600	0.28873400
N	3.49112900	-0.16307500	-0.18159200
C	3.69846000	-1.49437800	-0.77447700
H	3.77377000	-1.38369700	-1.86007800
H	4.63817100	-1.88719100	-0.38129500
H	2.88011200	-2.16285700	-0.51980200
C	4.68348900	0.70160000	-0.14691600
H	5.30749700	0.43057200	-0.99900100
H	4.40319000	1.75021900	-0.22610100
H	5.23183800	0.52029800	0.78239700
C	2.14444300	1.55422000	0.94637100
H	2.99071600	1.78884900	1.59633600
H	2.06569100	2.34158000	0.18642900
H	1.23206900	1.54947100	1.54417900

XYZ coordinate of BA-12

C	5.07652700	0.96318900	1.64872400
C	5.40997400	0.45787400	0.24478200
H	4.24533400	1.67709400	1.61021100
H	4.80716400	0.14270700	2.31661400
H	5.95028600	1.48000800	2.06245900
C	6.50122400	-0.61268000	0.25267700
C	5.81090200	1.62275400	-0.65954300
H	6.66912100	-0.99481200	-0.76121100
H	7.43854300	-0.16940400	0.60865100
H	6.23910700	-1.44699300	0.90636900
H	6.71767000	2.09873100	-0.27105300
H	6.01234000	1.27272300	-1.67825800
H	5.01477300	2.37465100	-0.70002700
O	4.19591700	-0.05410500	-0.41560200
C	3.50273300	-1.10852500	0.07689400
O	3.80822200	-1.72798300	1.09814600
C	1.93449600	-0.55144000	-1.78619400

C	1.44161100	-2.37799100	-0.22618300
C	0.71378900	0.23862500	-1.30186800
H	1.64796200	-1.20017400	-2.62531700
H	2.73506500	0.10480800	-2.12328000
C	0.20441700	-1.64197700	0.30153500
H	1.16008500	-3.02440300	-1.06860000
H	1.90430600	-2.99655700	0.54314300
C	-0.39045100	-0.68534100	-0.75099100
H	0.32565800	0.84141600	-2.13292200
H	1.03809900	0.93848000	-0.51882700
H	-0.54109800	-2.38498100	0.61375200
H	0.49046900	-1.07644800	1.19977800
H	-0.73745300	-1.30733100	-1.59390800
N	2.43908200	-1.42278400	-0.71729600
B	-1.63121900	0.16754900	-0.19647300
O	-2.29816700	1.03568800	-1.20633500
O	-1.30761700	1.07382300	0.93318200
O	-2.71210100	-0.79430400	0.41766300
C	-2.51172400	2.23453000	-0.60560700
C	-1.91814600	2.25579300	0.67240600
C	-3.40175000	-1.70888800	-0.16340300
C	-3.18470300	3.33872500	-1.10689800
C	-1.98325400	3.38520700	1.47564500
N	-4.02082100	-2.57246200	0.62739200
C	-3.52973800	-1.80164400	-1.65620500
C	-3.25423500	4.49087900	-0.29492000
H	-3.63880600	3.31515100	-2.09420200
C	-2.66464300	4.51338900	0.97166600
H	-1.52108800	3.39739800	2.45939000
C	-3.87738800	-2.44781400	2.08325100
C	-4.89721500	-3.64207000	0.14992700
H	-3.13388500	-2.75695600	-2.01626400
H	-4.58330800	-1.73888900	-1.94845700
H	-2.99353600	-0.98342000	-2.12943500
H	-3.77536500	5.37041000	-0.66457600
H	-2.72907200	5.41072000	1.58196800
H	-4.36707600	-1.53546000	2.43797400
H	-4.34878900	-3.31506200	2.54623400

H	-2.82086900	-2.41515700	2.35825400
H	-5.90432900	-3.48769300	0.55090600
H	-4.94342300	-3.65939700	-0.93593100
H	-4.51419200	-4.60269700	0.50765900

XYZ coordinate of BA-13

C	-4.04958600	2.28167500	-0.71622600
C	-4.55890500	1.22472500	0.26370400
H	-3.09314100	2.69243900	-0.37196300
H	-3.91741700	1.86914600	-1.71837300
H	-4.77372900	3.10298400	-0.76806400
C	-5.84612400	0.55402300	-0.21556900
C	-4.75804300	1.83459500	1.65045100
H	-6.14392700	-0.24148800	0.47766900
H	-6.65036800	1.29835800	-0.24473900
H	-5.72846700	0.12794000	-1.21387100
H	-5.52261300	2.61750700	1.60418200
H	-5.08518100	1.07282500	2.36704000
H	-3.82651900	2.28112200	2.01606900
O	-3.51461400	0.21190700	0.50845800
C	-3.03193400	-0.57193900	-0.48344600
O	-3.40539600	-0.52526600	-1.65659900
C	-1.48021300	-1.35287800	1.31125700
C	-1.30110800	-2.22111900	-0.97514500
C	-0.09813600	-0.69664400	1.21830500
H	-1.37801300	-2.37413800	1.70164000
H	-2.13845000	-0.79741900	1.97651500
C	0.08782100	-1.59719000	-1.14771200
H	-1.20696500	-3.24309400	-0.58482200
H	-1.84028100	-2.26084500	-1.92134400
C	0.80868800	-1.45178100	0.20888800
H	0.36174300	-0.68591400	2.21416000
H	-0.22304000	0.34819200	0.90209700
H	0.67980300	-2.21968300	-1.83050300
H	-0.02463500	-0.61220100	-1.61985100
H	0.97398900	-2.46046700	0.61987900

N	-2.09609500	-1.45001600	-0.01609300
B	2.19750400	-0.74908200	0.09769100
O	3.24863700	-0.92860700	1.00235300
O	2.55720300	0.17845500	-0.88324400
C	4.26316400	-0.09556000	0.57393900
C	3.84303500	0.57746700	-0.57450200
C	5.51861000	0.10087100	1.12370800
C	4.65435900	1.48412800	-1.23513500
C	6.35406600	1.01904700	0.46482900
H	5.83897200	-0.42709700	2.01664600
C	5.93173300	1.69514400	-0.68835800
H	4.31993500	2.00376400	-2.12785900
H	7.34847000	1.20604700	0.86017800
H	6.60377700	2.39824300	-1.17216900

XYZ coordinate of BB-1-DMF

C	2.88880500	-0.71433200	-0.51355900
C	2.67786800	0.29006400	-1.47549600
C	3.66749000	0.63412700	-2.38053000
C	4.89290300	-0.05423600	-2.30879000
C	5.10102100	-1.05018400	-1.35634100
C	4.09102100	-1.39617400	-0.43925900
H	3.50019100	1.40877500	-3.12190800
H	5.68479900	0.19821600	-3.00654100
H	6.05343000	-1.56887600	-1.31748700
H	4.24688800	-2.17182800	0.30353500
B	0.83529000	0.19238500	-0.14127600
C	-2.88916100	0.71400900	-0.51367500
C	-2.67804100	-0.29072400	-1.47521900
C	-4.09161500	1.39543800	-0.43943800
C	-3.66771300	-0.63556600	-2.37989800
C	-5.10167000	1.04865400	-1.35616000
H	-4.24763200	2.17135800	0.30304600
C	-4.89337100	0.05236300	-2.30821100
H	-3.50027300	-1.41048400	-3.12096300
H	-6.05426900	1.56700000	-1.31733600

H	-5.68531900	-0.20070500	-3.00568000
B	-0.83537400	-0.19191200	-0.14127500
O	1.42212000	0.78999600	-1.37019500
O	1.77519700	-0.88766200	0.24079200
O	-1.77546900	0.88804900	0.24040000
O	-1.42207900	-0.79014700	-1.36992000
O	1.07635300	1.30999800	1.02356100
C	0.63467100	2.48214800	0.83894600
N	0.66119300	3.41279000	1.76532400
C	1.18832800	3.16648000	3.10394700
H	2.01157700	3.85611800	3.30092300
H	0.39921100	3.33671900	3.83934000
H	1.54289700	2.14272500	3.17975500
C	0.14959000	4.75395200	1.49895900
H	0.94566700	5.48549100	1.65095600
H	-0.20745100	4.81803000	0.47287300
H	-0.67223500	4.97474900	2.18307300
O	-1.07638300	-1.30927500	1.02409200
C	-0.63463100	-2.48143200	0.83979400
N	-0.66054700	-3.41168100	1.76659900
C	-1.18712400	-3.16489300	3.10535100
C	-0.14915300	-4.75296300	1.50046100
H	-2.01133600	-3.85337700	3.30238200
H	-0.39810500	-3.33641100	3.84054300
H	-1.54020100	-2.14064100	3.18141900
H	-0.94520400	-5.48440800	1.65306900
H	0.20740700	-4.81741600	0.47422900
H	0.67297200	-4.97358400	2.18426900
H	0.21658800	2.76906700	-0.12306600
H	-0.21691700	-2.76870800	-0.12227200

XYZ coordinate of BB-2-DMF

C	1.98862800	-0.73737500	0.00187700
C	1.79418700	0.64108600	0.00054300
C	2.84317800	1.53520700	-0.00273500
C	4.12905200	0.97709800	-0.00480300

C	4.32563000	-0.40665900	-0.00364800
C	3.24681100	-1.30164100	-0.00023700
H	2.68144600	2.60637300	-0.00370500
H	4.98851600	1.63769700	-0.00740100
H	5.33527200	-0.80118700	-0.00547200
H	3.39214100	-2.37511400	0.00059500
B	-0.15295200	-0.34198500	0.00522600
O	0.42462300	0.90380500	0.00269500
O	0.74939000	-1.37291300	0.00509800
O	-1.51845600	-0.58245100	0.00762800
C	-2.40149800	0.39608900	0.00362000
H	-2.05931400	1.42469100	0.00815800
N	-3.65893000	0.14192700	-0.00543000
C	-4.21048100	-1.21909000	-0.00975100
H	-4.90131900	-1.29939500	-0.84837400
H	-3.41645000	-1.95188400	-0.10613000
H	-4.75225900	-1.36954800	0.92441000
C	-4.63397300	1.24201200	0.00128600
H	-5.26455500	1.13480300	0.88343100
H	-4.11559000	2.19730800	0.02399800
H	-5.24428600	1.16479900	-0.89791000

XYZ coordinate of BB-3-DMF

C	1.90021900	-0.77214000	-0.05331600
C	1.69771400	0.58571100	-0.33992000
C	2.68222200	1.52915500	-0.11062300
C	3.90106300	1.07639900	0.42538600
C	4.10386500	-0.27326100	0.71043700
C	3.09742100	-1.22575700	0.47196600
H	2.51787600	2.57765100	-0.33448900
H	4.69425900	1.79174800	0.61525100
H	5.05378700	-0.59914800	1.12096900
H	3.25027600	-2.27669200	0.69258700
B	-0.13907100	-0.55279700	-0.97451000
O	0.44775200	0.77324000	-0.85917300
O	0.79169200	-1.49501200	-0.37909600

O	-1.49183000	-0.66721600	-0.38985500
C	-2.24662600	0.33971100	-0.12761900
H	-1.86067800	1.34859600	-0.23244600
N	-3.50193700	0.19359800	0.22077300
C	-4.12330300	-1.11414200	0.41416500
H	-5.04247100	-1.15921100	-0.17165500
H	-3.44839700	-1.90245300	0.09466100
H	-4.36771800	-1.24512400	1.47092300
C	-4.33411800	1.36053000	0.50430100
H	-4.65934200	1.33077000	1.54607400
H	-3.76878700	2.27311100	0.32621000
H	-5.21155700	1.34422100	-0.14462000

XYZ coordinate of BB-1-Et_3N

C	-2.53333300	-0.24085400	-1.47062500
C	-2.86877000	-0.65800100	-0.17144100
C	-4.04569100	-1.33523700	0.08955300
C	-4.90477900	-1.59817400	-0.99578700
C	-4.57413600	-1.18613900	-2.28443100
C	-3.37350800	-0.49579300	-2.54082800
H	-4.29551700	-1.65330000	1.09634400
H	-5.83436600	-2.12985900	-0.82039600
H	-5.24843900	-1.39899100	-3.10775400
H	-3.11240000	-0.17429000	-3.54378600
B	-0.83911100	0.44019400	-0.05754600
C	2.67550600	0.06860400	-1.28569700
C	2.96324000	0.65558600	-0.04355400
C	3.61205000	0.05113700	-2.30413000
C	4.19034700	1.24015900	0.21092300
C	4.86387800	0.64672600	-2.05512300
H	3.38618700	-0.40462600	-3.26265000
C	5.14770700	1.22971000	-0.82226700
H	4.40474800	1.69371000	1.17312900
H	5.61499900	0.64908700	-2.83846900
H	6.11842800	1.68356800	-0.65065700
B	0.81511900	-0.20024300	0.05577200

O	-1.89034900	-0.31472100	0.69769700
O	-1.33871000	0.38687100	-1.47312000
O	1.41523900	-0.41867600	-1.29629700
O	1.89163500	0.56475400	0.77588500
C	-0.15504700	-2.73671600	0.33188000
H	-0.01148400	-3.68517400	0.84707300
H	-1.13157600	-2.36528000	0.61928000
C	-0.10118100	-2.95873100	-1.17158500
H	-0.83677300	-3.72813300	-1.42444100
H	-0.34227200	-2.05765300	-1.73091400
H	0.87602900	-3.31035400	-1.50817100
C	0.50421600	-1.42621100	2.30568500
H	1.19717500	-0.64287600	2.60528200
H	-0.49127300	-0.99208400	2.26805200
C	0.53182400	-2.54890900	3.33475000
H	0.12131400	-2.14814500	4.26657800
H	-0.08221200	-3.40662600	3.05396800
H	1.54180200	-2.89733300	3.55291600
C	2.25257600	-2.32212500	0.77130000
H	2.60637600	-2.10267800	-0.23107000
H	2.86268400	-1.74689000	1.46536200
C	2.45046800	-3.81678600	1.01034600
H	3.52784600	-4.00660800	0.98372400
H	2.08075900	-4.16871900	1.97132300
H	1.99949200	-4.42696400	0.22619400
C	-2.56176700	2.40796100	0.58338300
H	-3.07257100	1.93976700	-0.25563500
H	-2.67841100	3.48493400	0.45195200
C	-3.21894800	2.01166200	1.89830500
H	-2.85661400	2.60343200	2.74042700
H	-4.28972400	2.21460900	1.80106700
H	-3.09515900	0.95823300	2.13312700
C	-0.33382600	2.38554500	1.68109000
H	-0.74994800	1.71752400	2.43443800
H	0.69067000	2.07262700	1.52013600
C	-0.36676800	3.82929100	2.16621100
H	0.20382700	4.49514700	1.51566300
H	-1.37535100	4.23436600	2.26584400

H	0.09917700	3.86592000	3.15508900
C	-0.64176100	3.02215900	-0.70556500
H	-0.86518400	4.04021700	-0.38607800
H	-1.29352100	2.79690100	-1.54763000
C	0.80680700	2.92462100	-1.13776100
H	0.98494900	3.70539200	-1.88312700
H	1.50696200	3.08993300	-0.31903500
H	1.02893200	1.96870300	-1.60461300
N	-1.08089400	2.10101700	0.40806500
N	0.85135900	-1.75958600	0.87411100

XYZ coordinate of BB-2-Et_3N

C	-1.97143300	0.64242800	0.20406600
C	-1.94121500	-0.60474100	-0.40956400
C	-3.15256600	1.28399600	0.51336900
C	-3.08853200	-1.28870800	-0.75219800
C	-4.32872800	0.60344900	0.17210500
H	-3.16858100	2.25654200	0.98995000
C	-4.29772100	-0.65123900	-0.44506300
H	-3.05518500	-2.25984000	-1.23078700
H	-5.28469500	1.06451900	0.39255500
H	-5.22990900	-1.14546300	-0.69366200
B	0.11664400	0.05745600	-0.08257500
O	-0.66337100	1.06960300	0.41474800
O	-0.61218800	-0.98226400	-0.59248800
C	2.05311900	0.23980400	1.41159900
H	3.13850200	0.22740600	1.42613700
H	1.72132400	1.22222300	1.74397300
C	1.46991800	-0.85719000	2.28394900
H	1.85752500	-0.72504100	3.29643400
H	0.37932300	-0.80537600	2.35214900
H	1.74855300	-1.85725800	1.94958200
C	1.98136100	1.40764800	-0.82719700
H	1.49957000	1.30538400	-1.80096400
H	1.49118000	2.21437300	-0.28582500
C	3.45655400	1.70585000	-0.99997600

H	3.52201700	2.69848700	-1.45335200
H	3.99837600	1.74316400	-0.05454300
H	3.94957300	1.00668100	-1.67380300
C	2.20415000	-1.13181900	-0.73455800
H	1.53777400	-1.95438000	-0.48833200
H	2.11461100	-0.94051700	-1.80260700
C	3.62451400	-1.50976600	-0.35433200
H	3.90111100	-2.35465500	-0.99077000
H	4.35263000	-0.71997300	-0.52217100
H	3.70486200	-1.84628100	0.67942300
N	1.62659500	0.12082000	-0.05311300

XYZ coordinate of BB-3-Et_3N

C	-1.85464300	0.63057800	-0.25625200
C	-1.84629900	-0.76973900	-0.30897000
C	-2.97987300	1.33253100	0.13809400
C	-2.96470700	-1.51183300	0.02786200
C	-4.12098100	0.58643300	0.48379500
H	-2.97898100	2.41642400	0.17985200
C	-4.11365600	-0.80678300	0.42904100
H	-2.95270400	-2.59566200	-0.01432500
H	-5.01999800	1.10779800	0.79540200
H	-5.00705400	-1.36027400	0.69863600
B	0.17528900	-0.02667300	-1.02082200
O	-0.64436100	1.12052400	-0.64231800
O	-0.63081600	-1.21420000	-0.72837300
C	2.52370300	0.99276200	-0.74605600
H	3.46822700	0.88388100	-0.21748300
H	2.70281100	0.76343800	-1.79676300
C	2.01154100	2.41714100	-0.60477200
H	2.82192000	3.09080200	-0.89754900
H	1.15651500	2.61300400	-1.24923200
H	1.72910000	2.66743200	0.41884300
C	2.18202500	-1.44223700	-0.62113400
H	1.43169000	-2.17814600	-0.34256500
H	2.23859500	-1.42979300	-1.71058400

C	3.52690500	-1.83246100	-0.02861200
H	3.84015500	-2.75434000	-0.52738700
H	4.31072900	-1.09291400	-0.19711400
H	3.46840600	-2.04934100	1.03782600
C	1.33123600	0.01595600	1.24869400
H	0.53878200	0.75325500	1.36229600
H	0.92318800	-0.95022700	1.54318600
C	2.49513700	0.40517200	2.15074200
H	2.12402500	0.39073300	3.17999600
H	3.33938600	-0.27887300	2.09838300
H	2.85759600	1.41489500	1.95486200
N	1.59861000	-0.09063400	-0.24589000

XYZ coordinate of BB-4

C	6.56919800	0.77368700	1.31173100
C	6.40605400	0.76604300	-0.20529900
H	5.80327700	1.40086200	1.77621500
H	6.50187700	-0.22953200	1.72935200
H	7.54671800	1.19098100	1.56779700
C	7.39504600	-0.16828500	-0.89578100
C	6.52791100	2.18033400	-0.76221900
H	7.20744700	-0.19746100	-1.97267500
H	8.41159000	0.20337200	-0.74153000
H	7.33479400	-1.18140000	-0.50140400
H	7.52523700	2.57625300	-0.55762500
H	6.37079000	2.18814100	-1.84368900
H	5.79353000	2.84500100	-0.30042800
O	5.02103600	0.39985500	-0.56656700
C	4.49732500	-0.80221100	-0.25169500
O	5.08750700	-1.68497400	0.35511200
C	2.42935300	0.19994900	-1.22425300
C	2.40761700	-2.05725700	-0.28160500
C	1.46700700	0.70370600	-0.14076600
H	1.86226500	-0.14823100	-2.09352200
H	3.08928800	0.99562900	-1.55259300
C	1.44367000	-1.62484800	0.83042700

H	1.84584200	-2.43143200	-1.14304300
H	3.06349800	-2.85035900	0.06570500
C	0.60686900	-0.45311500	0.34755500
H	0.84546400	1.50526600	-0.54538400
H	2.04200500	1.10807600	0.69817800
H	0.79648000	-2.46082700	1.10781300
H	2.01657600	-1.32888500	1.71458300
H	-0.08226300	-0.77880100	-0.43101300
N	3.22042300	-0.92680800	-0.72692300
O	-0.20414400	-0.02541300	1.51039400
C	-1.34279900	0.60511700	1.36551100
O	-1.63715900	0.84066000	0.07439900
C	-2.88946300	1.34364500	-0.28213000
C	-3.93470500	0.46553100	-0.55094700
C	-3.03713600	2.71285600	-0.45189500
C	-5.15939200	0.97082700	-0.98006600
C	-4.26103700	3.21635300	-0.88373500
H	-2.19940500	3.36710300	-0.23980900
C	-5.31904600	2.34633800	-1.14367200
H	-5.97963900	0.29547000	-1.18887100
H	-4.38521900	4.28498500	-1.01611900
H	-6.27436100	2.73338800	-1.48032400
I	-3.66606600	-1.63668800	-0.30842800
S	-2.27212400	1.05196700	2.66186400

XYZ coordinate of BB-5

C	-5.91737100	0.19858100	2.05145900
C	-5.71509900	-0.94575100	1.06216000
H	-5.02623500	0.33052100	2.67129100
H	-6.13227500	1.13677300	1.54257400
H	-6.75571300	-0.03792200	2.71229900
C	-6.89576500	-1.10924100	0.10931300
C	-5.44406900	-2.24991900	1.80512500
H	-6.69024100	-1.89309800	-0.62499400
H	-7.78187300	-1.40446400	0.67769800
H	-7.11685800	-0.18305600	-0.41891000

H	-6.30928600	-2.51771600	2.41598900
H	-5.25377400	-3.06566300	1.10313600
H	-4.57709900	-2.15007000	2.46299000
O	-4.46701400	-0.74734500	0.30073900
C	-4.28736700	0.32002300	-0.50755100
O	-5.11327300	1.20668200	-0.68286900
C	-1.99294400	-0.63368500	-0.72960200
C	-2.60263800	1.45096800	-1.85643500
C	-0.98321300	0.09805200	0.15884500
H	-1.49868900	-0.98486500	-1.64202900
H	-2.41094200	-1.49874500	-0.22510600
C	-1.59672400	2.23798500	-1.00989600
H	-2.12758900	1.10984800	-2.78223500
H	-3.45864200	2.06511300	-2.12319100
C	-0.45191700	1.33782100	-0.55893400
H	-0.16096800	-0.57104200	0.41294300
H	-1.46833400	0.40227700	1.09316100
H	-1.20257900	3.07766600	-1.58985600
H	-2.10336600	2.64962400	-0.12979000
H	0.12551400	1.02420400	-1.43525600
N	-3.07570900	0.26877100	-1.13495900
O	0.41950100	2.13265900	0.27739800
C	1.74437500	1.77174300	0.28749900
O	1.86991100	0.66994300	1.22948800
C	3.07408200	0.04197200	1.32533000
C	3.58109000	-0.81360100	0.33879700
C	3.80248500	0.19084300	2.51072000
C	4.79252200	-1.47843000	0.52126100
C	5.00565100	-0.47898700	2.70516000
H	3.40183000	0.85163800	3.27099400
C	5.50808600	-1.31122100	1.70565200
H	5.17049200	-2.13379800	-0.25412800
H	5.55190300	-0.34580500	3.63292600
H	6.44759700	-1.83572200	1.84247400
I	2.49876200	-1.17442500	-1.47369100
S	2.94203700	3.02993800	0.30786800

XYZ coordinate of product of BB-5

C	6.42952700	0.61123600	1.42284900
C	6.31537300	0.72360300	-0.09467300
H	5.68673200	1.24798800	1.91127000
H	6.29045900	-0.41374700	1.76250600
H	7.42088100	0.94888900	1.73681400
C	7.27097900	-0.21568300	-0.82432300
C	6.53768000	2.16478000	-0.54077100
H	7.11695600	-0.15574700	-1.90525900
H	8.30173400	0.08223900	-0.61423300
H	7.13938000	-1.24917100	-0.50792500
H	7.54899100	2.48401200	-0.27860800
H	6.41723200	2.26077500	-1.62270900
H	5.82811600	2.83714700	-0.05213600
O	4.92450200	0.46921700	-0.52132200
C	4.32045900	-0.71823100	-0.30892500
O	4.84004600	-1.67679100	0.24557600
C	2.34570000	0.47844600	-1.25336000
C	2.15913300	-1.83214100	-0.46982200
C	1.39725200	0.96951700	-0.15239900
H	1.77421400	0.22647800	-2.15233800
H	3.06223200	1.24869200	-1.51787400
C	1.20646600	-1.41434400	0.65690000
H	1.58916200	-2.10565700	-1.36297200
H	2.75569800	-2.68978200	-0.17241700
C	0.45658400	-0.15848800	0.24725400
H	0.83256500	1.83307200	-0.51097600
H	1.98118900	1.28243000	0.71904700
H	0.49785500	-2.22022100	0.86388900
H	1.78132900	-1.22156700	1.56807900
H	-0.23828200	-0.38675500	-0.55981100
N	3.05305900	-0.72952100	-0.82290800
O	-0.34181800	0.24432600	1.42239600
C	-1.48101600	0.88429300	1.29304400
O	-1.73539400	1.23877800	0.03240500
C	-3.02369700	1.66967100	-0.34159000
C	-4.01762900	0.74990300	-0.57776000

C	-3.23565500	3.02702900	-0.55641900
C	-5.26469100	1.14344500	-1.02002300
C	-4.49062000	3.44192500	-1.00608500
H	-2.43899700	3.74014200	-0.37091200
C	-5.50315700	2.50931500	-1.23513700
H	-6.05124300	0.41724200	-1.20454400
H	-4.67423300	4.49710000	-1.17666800
H	-6.47671100	2.83850200	-1.58505000
I	-3.18289000	-2.19828800	-0.18648700
S	-2.42864400	1.23149700	2.61325100

XYZ coordinate of BB-6

C	5.28810300	0.29263100	1.79969400
C	5.16682400	1.06800400	0.49120500
H	4.40872900	0.46036800	2.42758000
H	5.39633700	-0.77665200	1.62575800
H	6.16560700	0.64506500	2.34832900
C	6.32809200	0.79535500	-0.46001500
C	5.04000600	2.56256400	0.76606800
H	6.17634000	1.31528500	-1.40995300
H	7.25438100	1.16958700	-0.01594400
H	6.44571300	-0.26900600	-0.65724900
H	5.94199300	2.92728900	1.26276500
H	4.91139600	3.12048300	-0.16479200
H	4.18458800	2.76803000	1.41446500
O	3.88255800	0.75557900	-0.16916000
C	3.58196400	-0.49024700	-0.58845400
O	4.30781200	-1.46607100	-0.45907700
C	1.39409000	0.57060900	-1.15127000
C	1.76317000	-1.82034100	-1.51890100
C	0.31798300	0.28230400	-0.09652600
H	0.93194600	0.66212200	-2.13944500
H	1.90483100	1.50244400	-0.93362000
C	0.69704400	-2.18248800	-0.47719900
H	1.31229500	-1.75801700	-2.51425300
H	2.54412200	-2.57477800	-1.54719300

C	-0.32772900	-1.06483300	-0.38940100
H	-0.42693000	1.08071100	-0.10584800
H	0.77778700	0.25612700	0.89631900
H	0.20910800	-3.12036900	-0.75476400
H	1.17343300	-2.32161400	0.49810900
H	-0.91584900	-1.02044200	-1.30582600
N	2.36419600	-0.52392300	-1.21099300
O	-1.24205800	-1.43951500	0.71343100
C	-2.45711700	-0.95655900	0.80374900
O	-2.76684700	-0.14781200	-0.22182200
C	-4.03622500	0.43636800	-0.29753700
C	-5.10068800	-0.22763400	-0.85122100
C	-4.21119400	1.75466500	0.11344300
C	-6.35048700	0.30971600	-1.01902700
C	-5.46502100	2.34767200	-0.04121200
H	-3.37873700	2.29617200	0.55056900
C	-6.52842300	1.63883800	-0.60010200
H	-7.16966800	-0.25252300	-1.45509300
H	-5.60912100	3.37342000	0.27869100
H	-7.49906100	2.10901900	-0.71699500
S	-3.45891800	-1.34015500	2.06803800

XYZ coordinate of transition state of BB-6

C	5.06003300	2.09553000	0.96909800
C	5.50389800	0.89181000	0.14315700
H	4.29131400	2.66280700	0.43710900
H	4.66643600	1.79532600	1.93878600
H	5.91491100	2.75731700	1.13166500
C	6.50839900	0.01345900	0.88287100
C	6.07406000	1.34452700	-1.19668900
H	6.75548300	-0.87083500	0.28899900
H	7.43059300	0.57756300	1.04600200
H	6.12576000	-0.30882400	1.84998700
H	6.96409800	1.95675300	-1.03442200
H	6.35598800	0.48538000	-1.81047500
H	5.34366700	1.94063800	-1.74941900

O	4.32992400	0.09267700	-0.26399300
C	3.52700900	-0.50464100	0.64003700
O	3.67241100	-0.44303800	1.85294000
C	2.20835200	-1.09546300	-1.39474000
C	1.43269700	-1.73562300	0.83564400
C	1.00383700	-0.16694400	-1.59073100
H	1.97376400	-2.09593600	-1.77219800
H	3.07134800	-0.72710800	-1.93877200
C	0.20289300	-0.82768300	0.70950700
H	1.19419400	-2.74405700	0.48329300
H	1.75495100	-1.80568400	1.87060600
C	-0.16625600	-0.66673700	-0.75909300
H	0.72769400	-0.13547000	-2.64801300
H	1.26984500	0.84774900	-1.27811300
H	-0.63094700	-1.25452300	1.27128000
H	0.43052500	0.15520300	1.13384100
H	-0.53552700	-1.61370800	-1.16329300
N	2.53639300	-1.22156500	0.02674200
O	-1.23847700	0.31988900	-0.89912200
C	-2.50203500	-0.06547200	-0.84292900
O	-3.33076600	0.98497500	-0.96600300
S	-3.15067900	-1.56221400	-0.43954700
C	-4.52523300	0.84530400	-0.22804400
C	-4.79462100	-0.37105700	0.35556700
C	-5.39083300	1.92433400	-0.11855200
C	-5.95927300	-0.57099400	1.07359500
C	-6.57397400	1.73015300	0.59920100
H	-5.15197700	2.88133000	-0.57055000
C	-6.86136700	0.49647300	1.18894100
H	-6.18187300	-1.52881700	1.53524100
H	-7.27442800	2.55228200	0.69770200
H	-7.78997100	0.36111300	1.73468900

XYZ coordinate of BB-7

C	-5.29455200	2.10471600	-0.44858600
C	-5.61538500	0.76748000	0.21271900

H	-4.50392100	2.62389200	0.10014900
H	-4.97817400	1.97710900	-1.48249200
H	-6.18564900	2.73798800	-0.43456000
C	-6.64924100	-0.03802300	-0.56837100
C	-6.07814100	0.98063100	1.64999000
H	-6.80872000	-1.01350600	-0.10070000
H	-7.60267000	0.49702800	-0.56440600
H	-6.34353900	-0.19016700	-1.60226900
H	-7.00088700	1.56524200	1.66130600
H	-6.27152500	0.02466500	2.14296900
H	-5.32267100	1.52092000	2.22592300
O	-4.38032800	-0.02305800	0.38980800
C	-3.64054900	-0.43323100	-0.66112900
O	-3.89827100	-0.19362400	-1.83278300
C	-2.12291200	-1.26494900	1.13778800
C	-1.52965300	-1.51510700	-1.22339600
C	-0.93479000	-0.32684300	1.37675100
H	-1.82534000	-2.30082600	1.33062700
H	-2.94552800	-1.02471900	1.80262200
C	-0.32277300	-0.58492000	-1.05742700
H	-1.22995600	-2.55460500	-1.05606600
H	-1.94226700	-1.43917400	-2.22544400
C	0.17350500	-0.63209300	0.38286800
H	-0.56673900	-0.44664700	2.39923700
H	-1.25746600	0.71191100	1.25202300
H	0.47331300	-0.88372300	-1.74448800
H	-0.61380300	0.44144300	-1.30411700
H	0.58670300	-1.62521600	0.59908000
N	-2.57245300	-1.18343200	-0.25367100
O	1.22870800	0.34530000	0.61481300
C	2.37070300	0.12029800	-0.04863000
O	3.16594900	1.24853200	-0.01085400
S	3.39205100	-1.37818600	0.36856900
C	4.50341200	0.95492700	-0.08075200
C	4.83551100	-0.39024300	0.10027400
C	5.47008800	1.92339000	-0.29682400
C	6.16726700	-0.79054600	0.06976900
C	6.80679700	1.51857200	-0.32338000

H	5.18742800	2.96004600	-0.43914200
C	7.15176900	0.17765500	-0.14152800
H	6.43563900	-1.83150200	0.20872800
H	7.58149500	2.25821400	-0.49147000
H	8.19381200	-0.12087400	-0.16760500

XYZ coordinate of transition state of BB-7

C	4.41436300	1.89231400	-1.36681000
C	5.06394900	0.81332700	-0.50539200
H	3.78228000	1.43992500	-2.13590600
H	3.80880300	2.57335200	-0.77115700
H	5.19412600	2.47088000	-1.86923200
C	5.88193900	1.39529400	0.64337700
C	5.92499700	-0.10775600	-1.36280700
H	6.27881000	0.59596300	1.27525800
H	6.72876900	1.95472800	0.23696300
H	5.28811300	2.06812800	1.26002800
H	6.73780600	0.46132400	-1.81969000
H	6.36268300	-0.90630700	-0.75862300
H	5.33304700	-0.56196900	-2.16129400
O	4.03503900	-0.10902300	0.01800400
C	3.05324300	0.31226000	0.83966500
O	2.90570500	1.46506900	1.21982600
C	2.25922800	-2.03461600	0.56889000
C	0.98764200	-0.41727400	1.89643500
C	1.13140300	-2.10936700	-0.48607800
H	2.10202300	-2.79774600	1.33649100
H	3.22776600	-2.20923600	0.11140200
C	-0.17349800	-0.43141900	0.87305600
H	0.81618900	-1.16926800	2.67136500
H	1.06586500	0.55652600	2.37253700
C	-0.16524500	-1.75194900	0.16622100
H	1.09843500	-3.11249200	-0.91838600
H	1.35815800	-1.39787700	-1.28721500
H	-1.11798800	-0.25305200	1.39281400
H	-0.01301900	0.38441500	0.16252500

H	-0.65130800	-2.56281100	0.70318500
N	2.25033800	-0.72759900	1.22944500
O	-1.36036900	-1.67094700	-1.27890200
C	-2.45002300	-1.07129700	-1.11223600
O	-2.48801800	0.32649100	-1.18268600
S	-3.81195100	-1.68987200	-0.09996300
C	-3.60032600	0.86326100	-0.59914700
C	-4.44689400	-0.04528900	0.04401500
C	-3.88539100	2.22002800	-0.63031000
C	-5.60543100	0.39470700	0.67387600
C	-5.04721100	2.66111400	0.00705500
H	-3.21823500	2.90900900	-1.13536500
C	-5.89783000	1.76044300	0.65197200
H	-6.26639900	-0.30576700	1.17137500
H	-5.28834100	3.71818500	-0.00403400
H	-6.79707800	2.11982600	1.13963900

XYZ coordinate of product of BB-7

C	3.21941000	-1.99215600	0.70211600
C	3.82368300	-0.59577800	0.81544500
H	2.30421500	-2.06287500	1.29448600
H	2.99151900	-2.24619800	-0.33102500
H	3.93085800	-2.72505200	1.09248300
C	5.07460500	-0.43198200	-0.04285600
C	4.12802500	-0.26447600	2.27258900
H	5.44392800	0.59598100	0.00942000
H	5.86105100	-1.09170000	0.33330900
H	4.88360800	-0.68364600	-1.08506200
H	4.86339600	-0.96808200	2.66958300
H	4.53382700	0.74589000	2.36658300
H	3.22405700	-0.33241500	2.88305900
O	2.82446700	0.42955900	0.45750900
C	2.32030200	0.52910300	-0.79201000
O	2.53141500	-0.27045700	-1.69441400
C	1.18729100	2.53630600	0.17586600
C	0.71804700	1.81115200	-2.11519400

C	-0.25029500	2.23880500	0.63556000
H	1.25316200	3.56602900	-0.18945500
H	1.89467200	2.41932800	0.99074300
C	-0.74490800	1.47587100	-1.77315000
H	0.79740600	2.85015900	-2.44951400
H	1.09529500	1.16614600	-2.90455600
C	-1.18716200	2.16347300	-0.52388100
H	-0.57395000	2.99452300	1.35796600
H	-0.23053500	1.28115200	1.17951300
H	-1.39266600	1.73138000	-2.61742400
H	-0.81028800	0.38346200	-1.65301400
H	-2.22205700	2.46531500	-0.40631800
N	1.56274600	1.65587100	-0.93217000
O	-4.09359200	1.14518300	2.17441300
C	-3.57471000	0.37910000	1.41509900
O	-2.35946900	-0.19249400	1.65700900
S	-4.25485500	-0.19517900	-0.14277300
C	-1.91842600	-1.01575100	0.64019100
C	-2.80410000	-1.16181700	-0.42510800
C	-0.68218400	-1.63499300	0.65974200
C	-2.47149300	-1.95909000	-1.51359300
C	-0.34738800	-2.43521900	-0.43347800
H	-0.00437000	-1.49558800	1.49285700
C	-1.23088100	-2.59755200	-1.50359800
H	-3.15230400	-2.07455900	-2.34849500
H	0.61525600	-2.93039700	-0.44992500
H	-0.94908300	-3.22138100	-2.34422500

XYZ coordinate of BB-8

C	-2.69480000	0.79292200	-0.00007200
C	-2.72986600	-0.60420000	0.00014300
C	-1.55195900	-1.35200400	0.00018300
C	-0.34257300	-0.66665300	0.00008600
C	-0.32080600	0.72638300	-0.00013200
C	-1.48014400	1.48101700	-0.00019900
H	-3.62117600	1.35563000	-0.00006200

H	-3.68378700	-1.11914800	0.00017900
H	-1.57890300	-2.43529300	0.00055900
H	-1.43757100	2.56375800	-0.00021500
O	0.93922500	1.29111000	0.00013300
C	1.95350500	0.37737700	0.00006500
O	3.10831100	0.69058500	0.00019900
S	1.30881300	-1.29672300	-0.00022200

XYZ coordinate of BB-9

C	2.48323500	-0.16700600	1.60521800
C	2.41108100	0.19812600	0.12521600
H	1.91338400	0.54841800	2.20459000
H	2.09640700	-1.16742900	1.79195500
H	3.52440700	-0.13008600	1.93681000
C	3.11850300	-0.82137900	-0.76281600
C	2.97922900	1.59422100	-0.10737200
H	2.99186500	-0.56404200	-1.81806800
H	4.18909000	-0.81411500	-0.54090700
H	2.73882200	-1.82870800	-0.59957300
H	4.03503900	1.61828700	0.17209400
H	2.89625400	1.87827000	-1.15943700
H	2.44791900	2.33577400	0.49445400
O	1.00431600	0.35247800	-0.29398200
C	0.12959000	-0.67529400	-0.24537500
O	0.40078100	-1.80324700	0.14512100
C	-1.48814300	1.08526800	-0.96155100
C	-2.23219100	-1.20816700	-0.52949200
C	-2.28618300	1.63439100	0.23396400
H	-2.11400200	1.09708000	-1.85929900
H	-0.60430000	1.68433300	-1.15653700
C	-3.07095000	-0.76970400	0.68443000
H	-2.84282000	-1.17132600	-1.43662600
H	-1.86030200	-2.22207100	-0.40671900
C	-3.37065800	0.69231900	0.64115900
H	-2.68752200	2.62272700	-0.01110500
H	-1.57756600	1.78511700	1.06776400
H	-3.98876200	-1.36379400	0.73512100

H	-2.49363400	-1.01330800	1.59404100
H	-4.26744700	1.07439200	1.11805200
N	-1.09695600	-0.30571900	-0.71942800

XYZ coordinate of BB-10-DMF

C	4.70006800	0.90934500	1.98486700
C	5.20189200	0.65839000	0.56540300
H	3.83347600	1.57621200	1.97232300
H	4.42127200	-0.01813000	2.48235100
H	5.48850800	1.39284500	2.56799900
C	6.35870800	-0.33627700	0.52320100
C	5.60487600	1.97234800	-0.09633700
H	6.64951600	-0.54212900	-0.51070800
H	7.22382600	0.09161700	1.03701900
H	6.09859300	-1.27594700	1.00775200
H	6.43020300	2.43004700	0.45394800
H	5.92985200	1.80597600	-1.12647200
H	4.76795000	2.67509900	-0.10685200
O	4.09586800	0.19548700	-0.29241100
C	3.44071100	-0.96254100	-0.04968900
O	3.69283800	-1.71934700	0.88043600
C	2.01395500	-0.18761500	-1.94529000
C	1.52036800	-2.27889900	-0.76894500
C	0.69943400	0.43266100	-1.46767600
H	1.85859300	-0.69383500	-2.90502600
H	2.78767900	0.56164700	-2.08405200
C	0.18840600	-1.72081700	-0.26038200
H	1.36923600	-2.79059000	-1.72613900
H	1.95246800	-2.99006700	-0.06873700
C	-0.37672900	-0.63448500	-1.19426000
H	0.34517400	1.14458600	-2.21996700
H	0.89531900	1.00599000	-0.55311200
H	-0.51766700	-2.55188800	-0.15463800
H	0.34579400	-1.30606000	0.74284900
H	-0.61109500	-1.11790400	-2.15643500
N	2.48249000	-1.19786500	-0.99006700
B	-1.71904600	0.02995800	-0.64600200

O	-2.34684200	1.03764500	-1.52451000
O	-1.62631300	0.67548800	0.68543400
C	-2.78290400	2.03415200	-0.71631300
C	-2.35462100	1.81994600	0.60546800
C	-3.52806700	3.15068200	-1.05429700
C	-2.66163000	2.71276800	1.61624500
C	-3.84393000	4.06383200	-0.03137700
H	-3.85533400	3.31438800	-2.07583100
C	-3.41945800	3.84954600	1.27857500
H	-2.32599900	2.54110900	2.63373800
H	-4.42758800	4.94669400	-0.27109000
H	-3.67452300	4.56598900	2.05254700
O	-2.82441700	-1.12369800	-0.52584900
C	-3.04242100	-1.71625900	0.57489700
N	-3.84963200	-2.74256300	0.68257000
H	-2.55758600	-1.36876400	1.48427400
C	-4.56586800	-3.29930600	-0.46208000
C	-4.07647000	-3.38568800	1.97450400
H	-4.30407800	-4.35355600	-0.56959700
H	-5.64064400	-3.21549400	-0.28931200
H	-4.29717400	-2.76091300	-1.36608800
H	-3.78101300	-4.43472500	1.91422100
H	-3.49031600	-2.88733900	2.74407800
H	-5.13621300	-3.32693700	2.22959100

XYZ coordinate of BB-10-Et_3N

C	-4.75012600	1.56111500	-0.98819200
C	-5.16058200	0.60696800	0.13039100
H	-3.84754200	2.11188700	-0.70922300
H	-4.56266300	1.03104900	-1.92054900
H	-5.54965700	2.28763500	-1.15666100
C	-6.36397600	-0.25223900	-0.24768500
C	-5.44176700	1.38286100	1.41327900
H	-6.59105700	-0.96534100	0.54974500
H	-7.23905100	0.38884900	-0.38492500
H	-6.18986500	-0.80256900	-1.17084200

H	-6.28075400	2.06569600	1.26059500
H	-5.69622000	0.70402800	2.23116100
H	-4.57009900	1.97128800	1.71050400
O	-4.02211100	-0.24576200	0.51640200
C	-3.43964400	-1.09751800	-0.35839200
O	-3.80807300	-1.26095300	-1.51542900
C	-1.79981700	-1.38662700	1.49615900
C	-1.49788400	-2.54013900	-0.63243500
C	-0.54241400	-0.55006900	1.26027600
H	-1.53792600	-2.30952000	2.02660900
H	-2.53074900	-0.85307200	2.09699900
C	-0.22870300	-1.73714200	-0.92912700
H	-1.24335900	-3.46463200	-0.10173000
H	-2.02142000	-2.80947500	-1.54717200
C	0.48410000	-1.27081000	0.35768800
H	-0.09194000	-0.30827500	2.22609000
H	-0.83934100	0.40429500	0.80704200
H	0.42480800	-2.36069100	-1.54248900
H	-0.49638900	-0.86880200	-1.53963800
H	0.77520800	-2.17919400	0.89663400
N	-2.40998800	-1.77757800	0.22120300
B	1.76325600	-0.29054000	0.10342400
O	1.93050300	0.73187200	1.18605800
O	1.60009900	0.52598300	-1.13768000
C	1.74600300	1.94635000	0.62406500
C	1.56316400	1.82598900	-0.76486500
C	1.74468500	3.18410500	1.24238400
C	1.38164700	2.94013400	-1.56444500
C	1.55354200	4.32232100	0.43546100
H	1.89134300	3.27173400	2.31393000
C	1.37651900	4.20282900	-0.94082400
H	1.24518800	2.84088200	-2.63633500
H	1.54563900	5.30432600	0.89722500
H	1.23082500	5.09213400	-1.54540500
N	3.24343500	-1.09059000	0.00843800
C	4.30286700	-0.27070300	-0.71217700
C	3.78485000	-1.39395200	1.38994000
C	3.12179800	-2.39331400	-0.75261800

C	4.88052200	0.93841900	0.00858100
H	5.11419800	-0.96642100	-0.93862200
H	3.85982000	0.04316000	-1.65142000
C	2.96963100	-2.32108800	2.27652500
H	4.78201500	-1.81321600	1.23524900
H	3.89575100	-0.43828000	1.89100700
C	2.94200700	-2.25262000	-2.25940600
H	4.02882700	-2.96539700	-0.54312800
H	2.28808700	-2.93904300	-0.32391100
H	4.13372600	1.68007300	0.28261600
H	5.45076300	0.67163800	0.89915000
H	5.57585000	1.41835600	-0.68648100
H	2.02484200	-1.87671000	2.58749400
H	2.76983100	-3.29309700	1.82329500
H	3.55905200	-2.50001700	3.18051500
H	2.17470000	-1.52707700	-2.52609400
H	3.86791100	-1.97008800	-2.76187900
H	2.63737100	-3.22458700	-2.65627600

XYZ coordinate of BB-11

C	-4.05675800	2.27623900	-0.72200200
C	-4.55624600	1.22200000	0.26192700
H	-3.10396300	2.69367400	-0.38474200
H	-3.92483100	1.86368300	-1.72087900
H	-4.78159600	3.09292000	-0.77687500
C	-5.84025100	0.54528700	-0.20925100
C	-4.75283000	1.83500000	1.64457000
H	-6.13287300	-0.24732700	0.48502600
H	-6.64795600	1.28160900	-0.23899000
H	-5.72706800	0.11631300	-1.20354800
H	-5.51826500	2.61327600	1.60189200
H	-5.07336700	1.07777100	2.36444600
H	-3.82511600	2.28571300	2.00593400
O	-3.50627000	0.21456800	0.50619900
C	-3.02826300	-0.57151200	-0.48352300
O	-3.40246000	-0.52685700	-1.64856000

C	-1.47565400	-1.34530300	1.31065900
C	-1.29798900	-2.21876900	-0.97168100
C	-0.09494100	-0.69254300	1.21619200
H	-1.37638800	-2.36206600	1.70657900
H	-2.13125200	-0.78904900	1.97351900
C	0.08950600	-1.59824900	-1.14558000
H	-1.20656600	-3.23936700	-0.58404800
H	-1.83517700	-2.26127000	-1.91589800
C	0.80981200	-1.44937800	0.20884200
H	0.36468400	-0.67908400	2.20928100
H	-0.21675300	0.35037700	0.90106700
H	0.67975000	-2.22032700	-1.82598400
H	-0.02032600	-0.61745000	-1.62108900
H	0.97521800	-2.45476000	0.62183300
N	-2.09197600	-1.44651000	-0.01476700
B	2.19475600	-0.75039200	0.09782600
O	3.24782000	-0.92927200	0.99815600
O	2.55488600	0.17818500	-0.87979900
C	4.25996500	-0.09622300	0.57124900
C	3.83857700	0.57770300	-0.57268600
C	5.51321100	0.10097000	1.11807300
C	4.64758000	1.48392200	-1.23033600
C	6.34544100	1.01890000	0.46200400
H	5.83538600	-0.42698500	2.00813800
C	5.92237600	1.69494800	-0.68623800
H	4.31315500	2.00468300	-2.12017400
H	7.33828300	1.20586700	0.85598200
H	6.59204900	2.39838100	-1.16851500

XYZ coordinate of BC-1

C	-4.81972400	0.00040000	-1.18365200
C	-3.43149800	0.00032000	-1.07536400
C	-2.83532200	-0.00006100	0.19505600
C	-3.63447600	-0.00035600	1.34833300
C	-5.02048800	-0.00027900	1.23159500
C	-5.61362200	0.00010000	-0.03401300

H	-5.28253900	0.00069600	-2.16578000
H	-2.81633400	0.00055200	-1.96739300
H	-3.15747600	-0.00064400	2.32241500
H	-5.63838700	-0.00051100	2.12440000
H	-6.69610100	0.00016200	-0.12437300
C	-1.37294400	-0.00015800	0.38477300
O	-0.77089400	-0.00036600	1.42915500
O	-0.70841600	0.00003100	-0.85028900
N	0.65829200	0.00005300	-0.71157700
C	1.36982900	1.18411300	-0.41977400
C	1.36989300	-1.18403800	-0.42007400
C	2.74376400	0.70098800	-0.11339000
O	0.90713300	2.30217100	-0.43956500
C	2.74380500	-0.70091400	-0.11358000
O	0.90727000	-2.30212100	-0.44016600
C	3.89666600	1.42389000	0.14972600
C	3.89675100	-1.42381900	0.14933400
C	5.06582800	0.69973200	0.41979500
H	3.89121000	2.50904300	0.14980300
C	5.06587000	-0.69966500	0.41960200
H	3.89136000	-2.50897200	0.14911900
H	5.98670700	1.23307700	0.63494900
H	5.98678000	-1.23301400	0.63461100

XYZ coordinate of BC-2

C	-4.74587100	0.00016400	-1.30389900
C	-3.36817100	0.00024600	-1.10182600
C	-2.85919700	0.00004200	0.20565500
C	-3.73410000	-0.00025100	1.30238900
C	-5.10913900	-0.00032800	1.09219900
C	-5.61557900	-0.00012000	-0.21050800
H	-5.14120100	0.00031900	-2.31510200
H	-2.69359400	0.00046200	-1.94984600
H	-3.32400200	-0.00041200	2.30650300
H	-5.78597100	-0.00055100	1.94124900
H	-6.68948900	-0.00018300	-0.37395000

C	-1.41257000	0.00010400	0.49313200
O	-0.87896800	-0.00031600	1.57212100
O	-0.67134100	0.00046700	-0.70147400
N	0.67581800	0.00028600	-0.49009500
C	1.39552700	1.21741300	-0.33502400
C	1.39524100	-1.21705900	-0.33538100
C	2.74689200	0.75140900	-0.12228900
O	0.90107300	2.33669600	-0.38128100
C	2.74671100	-0.75144000	-0.12249200
O	0.90049800	-2.33620300	-0.38189700
C	3.93481400	1.46186400	0.08849700
C	3.93446700	-1.46224000	0.08808800
C	5.09181400	0.74375900	0.28717500
H	3.93362600	2.54703400	0.09278300
C	5.09163500	-0.74446700	0.28697300
H	3.93301800	-2.54741000	0.09206700
H	6.03612800	1.25162600	0.45070100
H	6.03582500	-1.25260400	0.45037000

XYZ coordinate of BC-3

C	2.99904100	0.72275600	0.30507800
C	2.99904000	-0.72275600	-0.30508400
B	0.85038600	0.00000000	-0.00000100
C	-2.99904100	0.72276100	-0.30506700
C	-2.99904000	-0.72276100	0.30507300
B	-0.85038600	0.00000000	0.00000100
O	1.60965700	-1.13811400	-0.12328400
O	1.60965800	1.13811400	0.12328100
O	-1.60965700	1.13811600	-0.12326300
O	-1.60965700	-1.13811600	0.12326700
C	3.90269800	1.72192500	-0.40516400
H	3.81943100	2.70181700	0.07654400
H	4.94949200	1.40372900	-0.34669400
H	3.63146300	1.83693400	-1.45712800
C	3.26559000	0.74580400	1.81212800
H	4.31020600	0.50901700	2.03805000

H	3.04950900	1.74761900	2.19669700
H	2.62465800	0.03351100	2.34134200
C	3.90269900	-1.72192500	0.40515600
H	3.81943100	-2.70181700	-0.07655200
H	4.94949200	-1.40372900	0.34668400
H	3.63146600	-1.83693400	1.45712100
C	3.26558600	-0.74580400	-1.81213400
H	4.31020200	-0.50901700	-2.03805900
H	3.04950500	-1.74761900	-2.19670300
H	2.62465300	-0.03351100	-2.34134700
C	-3.90269900	-1.72191900	-0.40518200
H	-3.81943200	-2.70181800	0.07651000
H	-4.94949200	-1.40372300	-0.34670600
H	-3.63146600	-1.83691200	-1.45714900
C	-3.26558600	-0.74583200	1.81212300
H	-4.31020200	-0.50904800	2.03805100
H	-3.04950500	-1.74765300	2.19667600
H	-2.62465300	-0.03354700	2.34134700
C	-3.26559000	0.74583200	-1.81211600
H	-4.31020600	0.50904800	-2.03804200
H	-3.04951000	1.74765300	-2.19667000
H	-2.62465800	0.03354700	-2.34134200
C	-3.90269800	1.72191900	0.40519000
H	-3.81943200	2.70181800	-0.07650300
H	-4.94949200	1.40372300	0.34671600
H	-3.63146300	1.83691200	1.45715600

XYZ coordinate of BC-4

C	4.74861800	0.00116600	-1.34566800
C	3.37299200	0.00092900	-1.11342200
C	2.88755000	-0.00016700	0.20369300
C	3.78762400	-0.00106100	1.28003400
C	5.16083400	-0.00086000	1.04249000
C	5.64318800	0.00028200	-0.27088600
H	5.12157500	0.00204100	-2.36610400
H	2.68107200	0.00160000	-1.94775500

H	3.40068800	-0.00192300	2.29382900
H	5.85387400	-0.00157400	1.87930300
H	6.71419500	0.00046200	-0.45611900
C	1.43554300	-0.00036700	0.51641200
O	0.95040800	-0.00122700	1.62433800
O	0.68517200	0.00047200	-0.64083800
N	-0.67600000	0.00030400	-0.42492300
C	-1.41538400	-1.20096600	-0.29868300
C	-1.41548200	1.20138000	-0.29754800
C	-2.76336800	-0.72085500	-0.10633200
O	-0.91713200	-2.34352900	-0.36036700
C	-2.76345300	0.72098600	-0.10580000
O	-0.91733500	2.34404000	-0.35821100
C	-3.96817300	-1.42554700	0.06426800
C	-3.96832200	1.42543000	0.06536400
C	-5.14884300	-0.71296500	0.23175200
H	-3.96982200	-2.51325700	0.06509600
C	-5.14892800	0.71259900	0.23225600
H	-3.97005200	2.51313800	0.06714600
H	-6.08734600	-1.24581600	0.36554700
H	-6.08747700	1.24524500	0.36655300

XYZ coordinate of product of BC-4

C	5.11135000	-0.49491400	0.06566900
C	3.76116500	-0.84652900	0.07554400
C	2.77413000	0.13931400	-0.04476000
C	3.15572500	1.47960200	-0.17371100
C	4.50456400	1.83251100	-0.18119600
C	5.48624000	0.84495500	-0.06209600
H	5.87171200	-1.26618200	0.15862400
H	3.46161900	-1.88404500	0.17637200
H	2.37623400	2.22842600	-0.26605700
H	4.79126300	2.87648200	-0.28002300
H	6.53828100	1.11838800	-0.06838500
C	1.28672600	-0.19720800	-0.03887300
O	0.46434700	0.71635700	-0.17945700
O	1.05738600	-1.45608200	0.12207600

N	-1.12943500	-1.71765400	0.18831800
C	-1.57266100	-0.92986800	1.24859500
C	-1.65103100	-1.22234500	-1.00475400
C	-2.62858300	0.00555200	0.71994000
O	-1.21684100	-1.01203700	2.41639000
C	-2.67355800	-0.17066800	-0.66140300
O	-1.38261500	-1.60922300	-2.13417100
C	-3.46589500	0.90498000	1.36323400
C	-3.55576100	0.54684500	-1.45545000
C	-4.35857900	1.64074800	0.57125600
H	-3.42705600	1.03603400	2.44086300
C	-4.40214000	1.46498300	-0.81823300
H	-3.58482000	0.40471100	-2.53200900
H	-5.02654800	2.35948400	1.03859900
H	-5.10300200	2.05041800	-1.40749600

XYZ coordinate of transition state of BC-4

C	-4.27812100	-1.38599300	-1.29576800
C	-3.04227900	-0.93216000	-0.83219600
C	-2.98215400	0.12764100	0.08586600
C	-4.16998100	0.73100100	0.52574800
C	-5.40282600	0.28416600	0.04980800
C	-5.45997300	-0.77697600	-0.86036100
H	-4.31854800	-2.21561400	-1.99684600
H	-2.12464400	-1.40264500	-1.16794700
H	-4.11440300	1.54754000	1.23871400
H	-6.31847100	0.76056700	0.39066600
H	-6.42098400	-1.12869800	-1.22710200
C	-1.67979900	0.63819900	0.62826000
O	-1.60012200	1.29878300	1.65244900
O	-0.66172400	0.29037400	-0.16462000
N	0.77158700	0.40344400	0.57263200
C	1.59546300	1.26449700	-0.21663800
C	1.38669100	-0.86416400	0.73645600
C	2.90596900	0.59279600	-0.30537500
O	1.23159300	2.32451200	-0.70579000

C	2.74191000	-0.70986500	0.24019800
O	0.84291600	-1.84050600	1.30385000
C	4.11534300	1.03025700	-0.82554300
C	3.85860700	-1.56600300	0.32794900
C	5.21752900	0.16098900	-0.76606500
H	4.20922000	2.02651400	-1.25056300
C	5.07579500	-1.11835500	-0.18542000
H	3.76690000	-2.55437600	0.76932000
H	6.18332400	0.47631900	-1.15070300
H	5.94216500	-1.77520200	-0.14259500

XYZ coordinate of BC-5

N	2.11216100	0.00000000	-0.00015500
C	1.31446500	1.11457200	-0.00010600
C	1.31446500	-1.11457200	-0.00004200
C	-0.14363600	0.69717800	0.00002900
O	1.69464600	2.29469300	-0.00011400
C	-0.14363600	-0.69717800	0.00003700
O	1.69464600	-2.29469300	-0.00017200
C	-1.32742900	1.42190500	0.00009600
C	-1.32742900	-1.42190500	0.00010000
C	-2.53407500	0.70062600	0.00017100
H	-1.32466400	2.50894800	0.00008500
C	-2.53407500	-0.70062600	0.00017100
H	-1.32466400	-2.50894800	0.00010500
H	-3.48102100	1.23509100	0.00022500
H	-3.48102100	-1.23509100	0.00022900

XYZ coordinate of BC-6

C	1.83778100	-1.21407100	0.00240000
C	0.44606300	-1.21791200	0.00243700
C	-0.24669000	0.00013200	0.00010300
C	0.44607500	1.21811700	-0.00235000
C	1.83781200	1.21406300	-0.00243900
C	2.53127400	-0.00000500	-0.00006200

H	2.38264400	-2.15301800	0.00437400
H	-0.10576500	-2.15301700	0.00463200
H	-0.10569200	2.15323600	-0.00445000
H	2.38276600	2.15296500	-0.00444800
H	3.61755300	0.00005800	-0.00011700
C	-1.71200800	-0.00009100	0.00015400
O	-2.43868600	1.03950000	0.00644900
O	-2.43798300	-1.03970200	-0.00663000

XYZ coordinate of transition state of BC-6

C	1.89608300	1.21347900	0.01810100
C	0.49279400	1.23128000	0.01758000
C	-0.12170500	0.00002200	-0.00001100
C	0.49274800	-1.23125900	-0.01760100
C	1.89603800	-1.21350900	-0.01809500
C	2.58731000	-0.00002800	0.00000900
H	2.43701200	2.15578000	0.03157200
H	-0.06669500	2.15890100	0.02931900
H	-0.06677600	-2.15886000	-0.02935100
H	2.43693100	-2.15583100	-0.03155700
H	3.67330100	-0.00004900	0.00001700
C	-2.16531300	0.00000300	-0.00000200
O	-2.43011400	-1.16374400	0.04223100
O	-2.43007300	1.16376000	-0.04221600

XYZ coordinate of BC-7

C	1.21447900	-0.63275700	0.00005300
C	1.22722800	0.77256500	-0.00003900
C	0.00000000	1.39853000	0.00000000
C	-1.22722800	0.77256500	0.00003900
C	-1.21447800	-0.63275700	-0.00005300
C	0.00000000	-1.32438000	0.00000000
H	2.15469000	-1.17895300	-0.00001700
H	2.16413300	1.32305200	-0.00002900
H	-2.16413400	1.32305000	0.00002900
H	-2.15469000	-1.17895300	0.00001700
H	0.00000000	-2.41078700	0.00000000

XYZ coordinate of BC-8

C	-0.01097300	2.37365000	1.25527900
C	-0.01208500	3.73999400	1.22357900
C	0.00615800	4.45931600	-0.00032700
C	0.02120900	3.73966400	-1.22407400
C	0.01450500	2.37332100	-1.25547600
C	0.00066300	1.54301500	0.00000600
H	-0.01867100	1.84388700	2.20274700
H	-0.02310200	4.29227800	2.16136800
H	0.00837700	5.54464800	-0.00044400
H	0.03401500	4.29169200	-2.16199100
H	0.01970800	1.84332100	-2.20282500
B	-1.19791500	0.49790300	0.00927400
O	-1.36725400	-0.38387900	1.04680100
O	-2.08850200	0.32794700	-1.01252500
C	-2.60765100	-1.11337600	0.78924300
C	-2.78030400	-0.93770700	-0.76324400
C	-3.69843700	-0.41773100	1.60475700
C	-2.42704700	-2.55087600	1.25618900
C	-4.22021800	-0.80655700	-1.23766600
C	-2.03918900	-1.99759200	-1.58070300
H	-4.65729300	-0.93788500	1.51581800
H	-3.40577900	-0.41332500	2.65945600
H	-3.83365100	0.62061200	1.28568300
H	-2.27879800	-2.56998300	2.34085200
H	-3.31795100	-3.14486200	1.02479400
H	-1.56191200	-3.02360700	0.78553300
H	-4.23912700	-0.66442500	-2.32312200
H	-4.78526100	-1.71578900	-1.00486200
H	-4.72305900	0.04538600	-0.77439400
H	-2.51366300	-2.97945700	-1.48759700
H	-2.05499600	-1.70649900	-2.63569400
H	-0.99279900	-2.07837700	-1.27134300
B	1.19763500	0.49591900	-0.00917200
O	2.08873300	0.32559900	1.01211200
O	1.36508200	-0.38687900	-1.04614200
C	2.77883700	-0.94106300	0.76322100

C	2.60487600	-1.11757500	-0.78901200
C	2.03703300	-1.99949700	1.58193300
C	4.21924000	-0.81131500	1.23653300
C	2.42240900	-2.55518300	-1.25489600
C	3.69582600	-0.42363000	-1.60575400
H	2.51028800	-2.98198600	1.48920700
H	2.05388500	-1.70767200	2.63670400
H	0.99034000	-2.07931000	1.27332800
H	4.23909000	-0.66849100	2.32188100
H	4.78303700	-1.72137000	1.00392700
H	4.72276400	0.03972600	0.77234600
H	2.27339700	-2.57483600	-2.33944500
H	3.31285000	-3.14995200	-1.02373000
H	1.55710900	-3.02670900	-0.78333900
H	4.65417900	-0.94477500	-1.51718900
H	3.40240100	-0.41956100	-2.66024000
H	3.83241200	0.61476400	-1.28743600

XYZ coordinate of BC-10

C	0.41949000	-1.79641300	-1.40078800
C	1.14409000	-2.95413200	-1.50406200
C	1.58482300	-3.65680200	-0.35164500
C	1.21996500	-3.16826400	0.92830800
C	0.50134200	-2.01040500	1.07188400
C	0.10267900	-1.15693900	-0.08800900
H	0.08091900	-1.28063000	-2.29409200
H	1.37611900	-3.35462900	-2.48966700
H	2.15820500	-4.57347700	-0.45005600
H	1.50682800	-3.73402100	1.81328600
H	0.20357000	-1.67677500	2.06173900
B	-1.42877500	-0.80023200	0.01272600
O	-2.18871700	-0.35335500	-1.04241800
O	-2.17036200	-1.00848100	1.15159700
C	-3.58750100	-0.50722900	-0.66623400
C	-3.51753400	-0.51642700	0.90460400
C	-4.04826000	-1.84143400	-1.25918700

C	-4.38933300	0.63949600	-1.26789500
C	-4.50590100	-1.45651600	1.58433800
C	-3.60857200	0.87725600	1.52809700
H	-5.11213900	-2.02063000	-1.07385300
H	-3.88566400	-1.82325900	-2.34157700
H	-3.47594800	-2.67821000	-0.84608400
H	-4.34288800	0.58708600	-2.36075000
H	-5.44147200	0.57039500	-0.96989400
H	-4.00382700	1.61340000	-0.95936700
H	-4.37104000	-1.40997000	2.67020600
H	-5.53717200	-1.16298000	1.35897900
H	-4.36060400	-2.49238400	1.26930100
H	-4.62231800	1.28420900	1.45395100
H	-3.33940800	0.81121000	2.58697700
H	-2.91758100	1.57458200	1.04861500
B	0.90171200	0.35402300	-0.04076400
O	0.78140500	1.14281300	-1.23382800
O	0.53288100	1.16518800	1.08342600
C	0.45783500	2.49628200	-0.87108900
C	0.71482800	2.53803200	0.68877200
C	-1.00762800	2.74042700	-1.24499800
C	1.34517200	3.44208200	-1.68114800
C	-0.27517500	3.39389200	1.47535900
C	2.14075500	2.98370700	1.05291900
H	-1.30896400	3.77827000	-1.06370800
H	-1.14063400	2.52350400	-2.31010500
H	-1.66743800	2.07148500	-0.69364700
H	1.11674900	3.33247200	-2.74725800
H	1.16764200	4.48821400	-1.40534000
H	2.40642900	3.22116300	-1.54168900
H	-0.03185600	3.34854000	2.54289400
H	-0.22617300	4.44302500	1.16218600
H	-1.30023900	3.03998400	1.35089100
H	2.28491800	4.05317400	0.86495900
H	2.30596100	2.80167500	2.12035400
H	2.90317600	2.43565600	0.49512700
N	2.48664000	-0.07542100	0.08908800
C	3.00460100	-0.37083100	1.29742900

C	3.21108300	-0.29351800	-1.02567900
C	4.28176600	-0.89396500	1.43024800
H	2.36907500	-0.14401000	2.14263400
C	4.49210400	-0.82047600	-0.96486500
H	2.72886700	-0.01139000	-1.95170800
C	5.03692400	-1.13249900	0.28174200
H	4.67187300	-1.11070200	2.41854200
H	5.04905700	-0.98124800	-1.88127900
H	6.03784500	-1.54601600	0.35718400

XYZ coordinate of transition state of BC-10

C	0.56956600	-1.73464400	-1.47558900
C	1.48840800	-2.74624400	-1.64680200
C	2.00189800	-3.45449000	-0.53525500
C	1.54659400	-3.12856500	0.75558300
C	0.63960700	-2.10610600	0.94813900
C	0.15157400	-1.28746000	-0.15461200
H	0.16127900	-1.21761200	-2.33856600
H	1.80537700	-3.02253700	-2.65017500
H	2.71820400	-4.25772400	-0.67974300
H	1.90527100	-3.69790800	1.61040400
H	0.26040200	-1.90142500	1.94481700
B	-1.35461800	-0.89714500	-0.00755300
O	-2.15102100	-0.46587600	-1.04281800
O	-2.06086900	-1.12655400	1.14973000
C	-3.53457400	-0.68610600	-0.64026200
C	-3.43476700	-0.70139600	0.92983300
C	-3.94309600	-2.03717600	-1.23373900
C	-4.40214800	0.42371200	-1.21940700
C	-4.36063200	-1.69661500	1.61937900
C	-3.58768900	0.68040700	1.56468400
H	-4.99322000	-2.26781000	-1.02832000
H	-3.80374500	-2.00432500	-2.31899800
H	-3.32367200	-2.84861300	-0.83839900
H	-4.36787700	0.38409900	-2.31320300
H	-5.44491600	0.29603000	-0.90829700

H	-4.06516500	1.41368100	-0.90577600
H	-4.20987000	-1.64914900	2.70310500
H	-5.40959500	-1.45678700	1.41321600
H	-4.16597600	-2.72147500	1.29516200
H	-4.62255000	1.03395200	1.51169300
H	-3.29558900	0.62286600	2.61796600
H	-2.94280000	1.41377200	1.07700000
B	0.98581200	0.53405300	-0.03582500
O	0.80579100	1.26505000	-1.23468300
O	0.42185700	1.23848700	1.05960200
C	0.29454000	2.57431700	-0.89179300
C	0.47689600	2.63515900	0.67680800
C	-1.16754600	2.64232200	-1.33385000
C	1.10611300	3.61656400	-1.65942500
C	-0.61916700	3.38946400	1.41954600
C	1.84288400	3.19493500	1.10230500
H	-1.59081200	3.64031000	-1.17484500
H	-1.22477900	2.41075600	-2.40255300
H	-1.77115600	1.90429700	-0.80849700
H	0.93585000	3.49263900	-2.73457700
H	0.80155400	4.63331000	-1.38537600
H	2.17794700	3.51170400	-1.47447200
H	-0.43534300	3.33713900	2.49825600
H	-0.62815500	4.44598100	1.12871600
H	-1.60613200	2.96842400	1.22490800
H	1.91319700	4.27050400	0.90936900
H	1.97080300	3.03211600	2.17767600
H	2.66875900	2.69939600	0.58587400
N	2.45206700	0.09771800	0.15822400
C	2.89651200	-0.28686700	1.39077600
C	3.24977200	-0.10682500	-0.93388200
C	4.13070700	-0.88225100	1.55766400
H	2.23260300	-0.05831600	2.21293400
C	4.48495000	-0.70767600	-0.81808700
H	2.84612200	0.25105900	-1.87019700
C	4.93843000	-1.12703100	0.44093500
H	4.45107200	-1.16203000	2.55576200
H	5.08818500	-0.85111700	-1.70833900

H	5.90523200	-1.60718000	0.55047100

XYZ coordinate of BC-11+BC-12

C	0.19987300	-2.14214100	1.08822800
C	-1.08651000	-2.67860500	1.06446500
C	-1.73982600	-2.86481700	-0.15613700
C	-1.10576800	-2.50892900	-1.34873300
C	0.17879800	-1.96733400	-1.31848200
C	0.85535900	-1.77619600	-0.10117800
H	0.70507600	-1.99251300	2.03872400
H	-1.58601500	-2.94419200	1.99210900
H	-2.74761700	-3.26763800	-0.17576900
H	-1.62082800	-2.64155000	-2.29607800
H	0.66613000	-1.68180400	-2.24705000
B	2.27912800	-1.16038400	-0.06218900
O	2.93952500	-0.87229400	1.10652900
O	2.99829900	-0.84429400	-1.18788500
C	4.31824700	-0.55168900	0.75182200
C	4.18676200	-0.11494500	-0.75380300
C	5.12682300	-1.83715400	0.94050400
C	4.82292100	0.53301400	1.69383400
C	5.35447200	-0.51856700	-1.64410100
C	3.87980900	1.37350600	-0.92693400
H	6.19240100	-1.67173200	0.75299700
H	5.00900800	-2.18332700	1.97219400
H	4.77493100	-2.63122900	0.27415700
H	4.86179100	0.14380500	2.71656300
H	5.83393000	0.84753300	1.41268500
H	4.17051100	1.40914400	1.68902300
H	5.16506100	-0.18937400	-2.67117100
H	6.28090000	-0.04435200	-1.30211800
H	5.49992900	-1.60114700	-1.65576500
H	4.75309100	1.99030400	-0.69205100
H	3.59634600	1.56096300	-1.96731400
H	3.05071900	1.68589200	-0.28667100
B	-2.46971500	0.99664900	0.05776500

O	-2.07843600	1.65013100	1.19896400
O	-1.81325400	1.38688300	-1.08105400
C	-0.89846600	2.43447500	0.84380700
C	-1.03075900	2.56533000	-0.72416800
C	0.31967600	1.62333500	1.28138500
C	-0.95515800	3.75457000	1.60091200
C	0.28902100	2.50209600	-1.48163000
C	-1.84651900	3.78204000	-1.16401300
H	1.24840900	2.17725300	1.11429200
H	0.23963400	1.40368300	2.35077500
H	0.37540800	0.67406100	0.74735500
H	-0.87362000	3.56433400	2.67621500
H	-0.12123800	4.40160500	1.30786000
H	-1.89151700	4.28721700	1.41979100
H	0.10108600	2.59752500	-2.55626900
H	0.94974200	3.32158400	-1.17944000
H	0.80251100	1.55394300	-1.31150000
H	-1.30338500	4.71558500	-0.98640900
H	-2.05249100	3.70308300	-2.23615800
H	-2.80499000	3.83159100	-0.63721500
N	-3.50660200	0.00360500	0.04882700
C	-4.00386100	-0.51643500	-1.16150300
C	-4.04571700	-0.49986900	1.24814300
C	-4.96921300	-1.48142900	-1.17750300
H	-3.56248400	-0.09720400	-2.05544500
C	-5.00640500	-1.46984000	1.24297400
H	-3.63218500	-0.07238100	2.15169100
C	-5.50662600	-2.00420300	0.02727600
H	-5.31649700	-1.84346100	-2.14110000
H	-5.38148200	-1.82494900	2.19868100
H	-6.27379300	-2.76949300	0.01932500
